# Support amongst UK pig farmers and agricultural stakeholders for the use of food losses in animal feed

**DOI:** 10.1371/journal.pone.0196288

**Published:** 2018-04-24

**Authors:** Erasmus K. H. J. zu Ermgassen, Moira Kelly, Eleanor Bladon, Ramy Salemdeeb, Andrew Balmford

**Affiliations:** 1 Conservation Science Group, Department of Zoology, University of Cambridge, Cambridge, United Kingdom; 2 Wildlife Population Health Group, University of Gent, Gent, Belgium; 3 Department of Zoology, University of Cambridge, Cambridge, United Kingdom; 4 Department of Engineering, University of Cambridge, United Kingdom; University of Illinois, UNITED STATES

## Abstract

While food losses (foods which were intended for human consumption, but which ultimately are not directly eaten by people) have been included in animal feed for millennia, the practice is all but banned in the European Union. Amid recent calls to promote a circular economy, we conducted a survey of pig farmers (n = 82) and other agricultural stakeholders (n = 81) at a UK agricultural trade fair on their attitudes toward the use of food losses in pig feed, and the potential relegalisation of swill (the use of cooked food losses as feed). While most respondents found the use of feeds containing animal by-products or with the potential for intra-species recycling (i.e. pigs eating pork products) to be less acceptable than feeds without, we found strong support (>75%) for the relegalisation of swill among both pig farmers and other stakeholders. We fit multi-hierarchical Bayesian models to understand people’s position on the relegalisation of swill, finding that respondents who were concerned about disease control and the perception of the pork industry supported relegalisation less, while people who were concerned with farm financial performance and efficiency or who thought that swill would benefit the environment and reduce trade-deficits, were more supportive. Our results provide a baseline estimate of support amongst the large-scale pig industry for the relegalisation of swill, and suggest that proponents for its relegalisation must address concerns about disease control and the consumer acceptance of swill-fed pork.

## Introduction

Food losses, i.e. foods which were intended for human consumption, but which ultimately are not directly eaten by people [1], have long been used as an animal feed–they have, for example, been fed to pigs since the very domestication of wild pigs, around 10,000 years ago [2]. While food losses continue to be included in animal feed in many parts of the world, the use of food losses in animal feed was all but banned in the European Union (EU) in 2002, after the 2001 foot-and-mouth outbreak, which is thought to have been started by a farmer illegally feeding uncooked food waste to pigs in the UK [3].

Current EU legislation permits the inclusion of only a small subset of food losses in animal feed. For example, all food losses containing animal by-products (materials of animal origin that people do not consume e.g. tendons, processed animal proteins) are banned, except for those containing honey, eggs, pig or poultry gelatine, milk products, rendered fats, and collagen, where there is no risk of contamination with other sources of animal by-products [4]. These legal food losses are known as former foodstuffs. The legislation specifically bans catering wastes (i.e. food that has been through a home kitchen or restaurant, making up the 57% of food losses in the EU [5]) and feeds where there is the potential for intra-species recycling–i.e. pigs eating pork products, or chickens eating poultry products.

These regulations deliver a safe food system to millions of Europeans, though they are not without their trade-offs. The current legislation limits the potential for nutrient recycling and a circular economy–food losses that are not used as feed are instead disposed of in less efficient ways, lower down the food waste hierarchy [6]. Recent studies have shown that the relegalisation of food losses in animal feed could cut feed costs for pig producers, reduce the land use of EU pork production by 22% (1.8 Mha), and reduce a host of other environmental pressures [7]. The ban on animal by-products in feed also treats all livestock in Europe as being essentially vegetarian, though, of course, pigs and poultry are omnivorous.

In light of these trade-offs and the existence of regulated systems for incorporating food losses in feed in other countries, there have therefore been intermittent calls to relegalise the use of food losses in feed [2,8–10]. Japan and South Korea, for example, operate systems for safely recycling food losses as animal feed, based on the heat-treatment of food losses (heat-treated food losses are colloquially known as “swill”, though they are marketed as “Ecofeed” in Japan [11]). Heat-treatment disactivates pathogens (such as foot-and-mouth) in the food, renders it safe for use as animal feed, and facilitates these countries recycling ca. 40% of their food losses as animal feed, compared with the 3–6% achieved in the EU [11,12].

Still, the debate continues to be polarised, with some arguing that the use of swill is unsafe or unnatural–the UK retailer The Co-operative, for example, banned the use of swill in 1995 [13]—while others argue that the ban was an exaggerated response to a manageable risk [14]. Little work has been done, however, to determine the attitudes of the people most affected by the ban on the use of food losses as feed–namely, pig farmers and workers in the agricultural sector. We therefore conducted a survey to investigate the attitudes of the farming community to the use of food losses as feed.

## Method

The survey was conducted at the British Pig & Poultry Fair on the 10-11^th^ May 2016 at Stoneleigh, Warwickshire. This fair was selected because it is the largest industry fair in the country dedicated to the pig and poultry industries, with 10,000 attendees visiting 350 stands from businesses and organizations involved in the sector. Fifty-eight percent of visitors were pig or poultry producers, with the remaining 42% made up of traders, advisors, students, processors, veterinarians, retailers, and others [15].

Visitors were invited to complete a survey at a University of Cambridge stand. Pairs of survey workers were also positioned at the entrance to the exhibit building to invite visitors to complete the survey as they entered. To incentivize completing the survey, visitors were offered food and drink at the stand, and people completing the survey were offered the chance to enter a raffle to win one of five £50 prizes. The survey was offered in both an electronic format (using the survey software Qualtrics, available on a tablet), or on paper.

Participants were assured that their responses were anonymous, and the study received ethical approval from the Ethics Committee for the School of the Humanities and Social Sciences, University of Cambridge, prior to be being conducted.

### Survey structure

The survey consisted of 18 sets of questions on eight themes, described below. A copy of the survey is available online (S1 Appendix).

(i) Respondents were asked about the acceptability of using ten different sources of food losses in pig feed (Table 1). These ten different sources of potential animal feed were selected for inclusion in the survey because they represent a range of combinations of legality, of whether or not they contain animal by-products, and of their potential for intra-species recycling. Respondents were asked: “How would you feel about the inclusion of the following in pig feed”, and scored each feed on a 1–5 Likert scale, from “1 = very uncomfortable” to “5 = very comfortable”. To check for the internal consistency of these constructs, the questions were repeated using two other Likert scales, “1 = Very negative” to 5 = Very positive” and “1 = Very dissatisfied” to “5 = Very satisfied”. The order that each feed and each Likert scale was presented was randomized, and respondents were also given a “Don’t know” option.

**Table 1:** Characteristics of different sources of food losses.

Food losses to be used as feed	Permitted under current legislation?	Potentially containing animal by-products?	Potentially entailing intra-species recycling?
Heat-treated restaurant leftovers	No	Yes	Yes
Biscuit crumbs from biscuit factories	Yes	No	No
Unsold confectionery containing porcine gelatine	Yes	Yes	Yes^a^
Unsold bread from supermarkets	Yes	No	No
Unsold egg sandwiches from supermarkets	Yes	Yes	No
Heat-treated leftovers from a college canteen	No	Yes	Yes
Misshapen chocolates from chocolate factories	Yes	No	No
Heat-treated, unsold bacon sandwiches from supermarkets	No	Yes	Yes
Heat-treated, unsold chicken sandwiches from supermarkets	No	Yes	No
Heat-treated household food leftovers	No	Yes	Yes

Food losses are listed by their legality, whether or not they contain animal by-products, and whether there is the potential for intra-species recycling.

^a^Gelatine products are exempt from the ban on intra-species recycling.

(ii) Respondents were asked how they thought heat-treated swill and conventional grain- and soybean-based feed compare in terms of eight different attributes, each scored on a five-point Likert scale (e.g. swill is: “1 = Much less nutritious” to “5 = Much more nutritious”, “1 = Much lower disease risk” to “5 = Much higher disease risk”, or “1 = Much lower cost” to “5 = Much higher cost”). The order in which each attribute was presented was randomized, and respondents were also given a “Don’t know” option.

(iii) Respondents were asked how they thought the performance of pigs reared on swill would compare with pigs reared on conventional diets. Four attributes (growth rates, feed conversion ratios, environmental impacts, and feed costs) were scored on a 5-point Likert scale (with a “Don’t know” option), and their order was randomized.

(iv) Respondents were asked how they believed pork from pigs reared on a diet containing swill would compare with pigs fed conventional diets. Six attributes (e.g. fattiness, tastiness, and marketability) were scored on a 5-point Likert scale, including a “Don’t know” option. The order of each attribute was randomized.

(v) Respondents were then asked to what degree they agreed with two statements: that swill is either a “unnatural practice” or a “traditional practice”. Both questions were scored on a 5-point Likert scale (“1 = Definitely not” to “5 = Definitely yes”), and the order of their presentation was randomised.

(vi) The next question was: “If the procedures were put in place to ensure the safety of swill (e.g. heat treatment was performed by regulated swill manufacturers), would you support the relegalisation of swill?”, and respondents reported their attitude on the same 5-point Likert scale.

(vii) Respondents were then asked to reflect on the values underlying their position on the relegalisation of swill, indicating how important twelve different issues were to them (e.g. food safety, perception of the pork industry, meat quality, environmental impacts etc.). The importance of these issues was scored on 5-point Likert scale (“1 = Not at all important” to “5 = Very important”), and the order of their presentation was randomized. Similarly, respondents were asked to agree/disagree with 12 statements about the impacts of using heat-treated swill (e.g. swill would “Lower dependence on foreign protein sources”, “Lower consumer acceptance of pork products”, or “Increase the risk of toxins entering the feed”. Their agreement was scored on a 5-point Likert scale, “1 = Totally disagree” to “5 = Totally Agree”, with a “Don’t know” option.

(viii) Finally, respondents were asked about their general characteristics (age, job, gender etc.), and pig farmers were asked about their farming practices. These included questions about the number of pigs, whether they use wet or dry feeds, whether they have previously used swill on their farm, whether their farm was affected by the 2001 foot-and-mouth outbreak, and whether they would consider using swill on their farm, if the use of swill were legalised.

The options included in each question on the comparative performance of swill and its perceived impacts were based on literature on the use of swill and the use of novel animal feeds [11,16]. Prior to the fair, the survey was piloted and refined, to ensure that questions were relevant and easily understood, and that the survey software worked smoothly.

### Details of survey respondents

Across the two days of the fair, 163 people completed the survey, including 82 pig farmers (13 farmers both with pigs and poultry and 69 farmers who keep only pigs). The 81 non-pig farmers included a variety of professions associated with the livestock industry, including poultry farmers, agricultural advisors, traders, and veterinarians (Figure A in S2 Appendix). Since our sample included only six respondents who reported not being directly employed in the animal industry (of which one was a former pig farmer) and given that their attendance at an agricultural trade fair suggests a strong interest in farming, for the purposes of analysis, we grouped all respondents into one of two groups: pig farmers (including farmers both with pigs and poultry) and other agricultural stakeholders.

Amongst pig farmers, 73% (60/82) of our sample were farmers with more than 1,000 pigs–our sample therefore captures views within the mainstream pork production industry. Though there are many small farms (<100 pigs) in the UK (Figure B in S2 Appendix), these host only 2% of the national herd, and therefore represent only a small market share [17], often for local consumption.

The median age group of respondents was 31–50 (Figure A in S2 Appendix), with 142 men and 21 women. Overall, 158 surveys were completed on tablets, and 5 completed on paper. Surveys took a median of 18 minutes to complete.

### Statistical analysis

All statistical modelling was done in R version 3.1.1 [18]. The internal reliability of the three constructs about the acceptability of different sources of food losses as pig feed (from (i), above) was tested by calculating their Cronbach alpha. Values exceeding 0.80 indicate a good degree of internal reliability. To understand the differences in acceptability of different sources of food losses as pig feed (i.e. why some are more acceptable than others), an ordered categorical model with a cumulative link was fit to the data. This modelled the acceptability of each feed as an ordered categorical variable (from “1 = Very unacceptable” to “5 = Very acceptable”), as a function of several predictors, including the respondent’s characteristics (e.g. job, gender from (viii), above), and characteristics of the feed (e.g. its legal status, as listed in Table 1) with varying intercepts for each feedstuff and respondent. The models were fit with Bayesian methods using weakly informative priors and the “rethinking” package [19]. We ran seven different models, with different predictors included in each (Table 2); these models were compared on the basis of their Widely Applicable Information Criterion (WAIC) score, an information criterion which makes no assumptions about the shape of the posterior distribution [20], and predictions were made using model averaging. Model fitting occurred in two stages. First, models were fit and de-bugged using three chains, each 4,000 iterations long, including a 2,000 iteration warm up. Once we were satisfied that each model had successfully converged (by checking chains, the effective number of samples, and ensuring the Gelman-Rubin convergence diagnostic, Rhat <1.01), a single longer chain (10,000 iterations with a 5,000 warm up iterations) was fit and used for parameter estimation, plotting, and prediction. The equations of these models are listed in the S2 Appendix.

**Table 2:** Models explaining the acceptability of different feeds, listed in order of their Akaike weights.

	Predictors							Model output		
Model ID	Respondent intercepts	Feed intercepts	Feed:job interaction	Slope for feed legality	Slope for animal by-products	Slope for intra-species recycling	Job:legality interaction	WAIC	pWAIC	Model weight
AC1	Y	Y	Y	Y	Y	Y	Y	10337.5	182.7	0.28
AC2	Y	Y	Y	Y	Y	Y	-	10338.1	183.3	0.20
AC3	Y	Y	Y	Y	Y	-	-	10338.8	183.7	0.14
AC4	Y	Y	Y	Y	-	Y	-	10338.9	183.6	0.14
AC5	Y	Y	Y	-	Y	Y	-	10339.0	183.8	0.13
AC6	Y	Y	Y	-	-	-	-	10339.3	183.9	0.11
AC7	Y	Y	-	Y	Y	Y	-	10353.4	176.5	0.00

WAIC is the widely applicable information criterion score, pWAIC is the number of effective parameters, and model weights are the Akaike weights. Model equations are listed in S2 Appendix. Models AC2-AC6 have sufficiently similar WAIC scores that their relative weighting differs between model runs; the results reported here are representative of typical results.

To understand each respondent’s position on the relegalisation of swill, we also fit Bayesian ordered categorical models to predict both support for relegalisation (9 models fit to data from all respondents and 14 models fit to data from pig farmers only, respectively listed in Table 3 and Table A in S2 Appendix) and farmer willingness to use swill on their farm (19 models, Table B in S2 Appendix). Predictor variables included information about the respondent’s characteristics (age, job, gender etc.), and the characteristics of the farm (number of pigs, whether or not the farm was affected by the 2001 foot-and-mouth outbreak etc.). We also used factor analysis to simplify the responses to the 12 questions about the importance of different issues (e.g. food safety) and the impact of swill (e.g. to what degree they (dis)agreed that swill would “lower dependence on foreign protein sources”) into a smaller number of factors, which were also included as predictor variables. The equations of these models are listed in the S2 Appendix.

**Table 3:** Models predicting support for the relegalisation of swill, amongst all respondents (n = 163).

	Predictors								Model output		
Model ID	Age group intercepts	Gender	Job	Job:gender interaction	Respondent's values: 1st factor loading	Respondent's values: 2nd factor loading	Respondent's perception of swill: 1st factor loading	Respondent's perception of swill: 2nd factor loading	WAIC	pWAIC	Model weight
AR1	Y	Y	Y	Y	Y	Y	Y	Y	342.9	13.4	0.12
AR2	Y	Y	Y	-	Y	Y	Y	Y	345.9	12.1	0.03
AR3	-	Y	Y	Y	Y	Y	Y	Y	340.6	11.8	0.39
AR4	-	Y	Y	-	Y	Y	Y	Y	344.2	10.7	0.06
AR5	Y	Y	-	-	Y	Y	Y	Y	344.0	11.1	0.07
AR6	Y	-	Y	-	Y	Y	Y	Y	347.1	10.6	0.02
AR7	-	-	Y	-	Y	Y	Y	Y	345.3	9.6	0.04
AR8	-	Y	-	-	Y	Y	Y	Y	342.2	9.7	0.17
AR9	-	-	-	-	Y	Y	Y	Y	343.1	8.4	0.10

Models are listed in order of their Akaike weights. WAIC is the widely applicable information criterion score, pWAIC is the number of effective parameters. Model equations are listed in S2 Appendix.

Factor analysis was done using the “psych” package [21] with polychoric correlation, as recommended for ordinal data [22]. We selected the number of factors on the basis of the Minimum Average Partial correlation [23]. We identified two factors which explained 45% of the variance in the response about the respondent’s values (i.e. how important different issues were to them). The first factor combined concerns about disease control and the perception of the pork industry by consumers (i.e. factor 1 had factor loading >0.5 for communication with consumers, traceability, perception of the pork industry, labelling of the end product, food safety and disease control). The second factor identified concerns about financial performance and efficiency (factor loading >0.5 for feed prices, profitability, and efficient use of resources).

Similarly, we identified two factors which explained 43% of the variance in respondents’ perception of the impact of swill. The first factor grouped perceptions that swill would benefit the environment, help farms financially, and reduce trade-deficits (factor loadings > 0.5 for reduce the environmental impact of food waste disposal, reduce the environmental impact of pork production, be an efficient way to use food waste, help farms reduce feed costs, help farmers improve profitability, and lower dependence on foreign protein sources). The second factor grouped perceptions that swill would increase disease risk and be unpalatable to consumers (factor loadings > 0.5 for increase the risk of prion diseases like bovine spongiform encephalopathy, reduce traceability, negatively affect the marketability of pork, increase the risk of toxins entering the feed, increase the risk of an outbreak of foot-and-mouth disease, and lower consumer acceptance of pork products). Missing values (“don’t knows”) were imputed as the median value [21].

Parameter estimates from the Bayesian models were converted into estimates of their effect size (e.g. how much support for the relegalisation of swill differed between farmers who used wet feeding systems versus those using dry feeding systems), by simulating the responses of 1,000 respondents in each group (e.g. wet feeders vs dry feeders), taking into account both parameter uncertainty and sampling uncertainty. Parameter uncertainty was included by sampling from the model averaged posterior distributions, and sampling uncertainty was accounted for by modelling responses using an ordered categorical probability density function. The data and code used for all analyses are available in the supplementary material (S2 Appendix).

## Results

### Acceptability of use of different sources of food losses in animal feed

**Fig 1:**
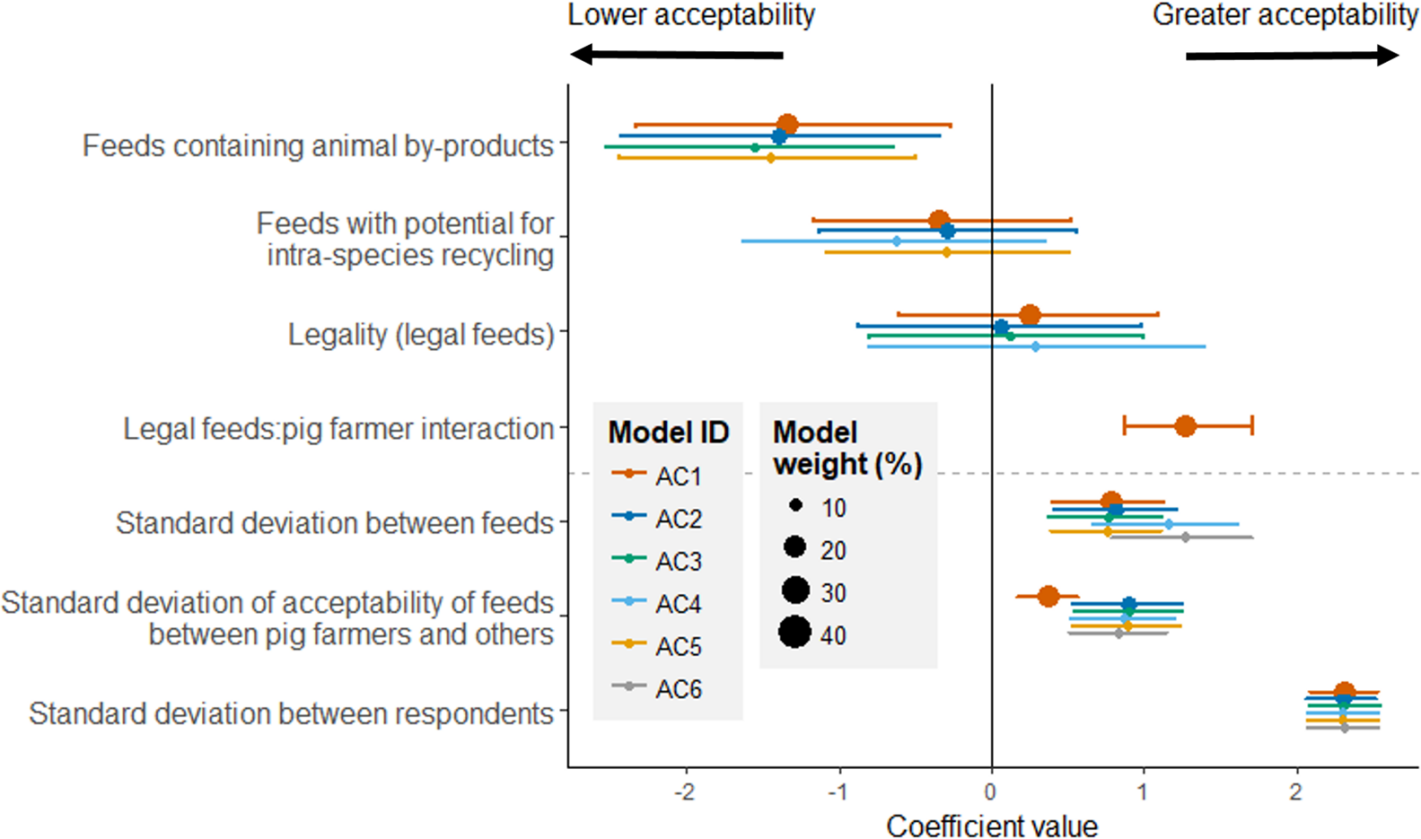
Estimates from the six models with the greatest weighting (summing to 100% of model weight) of how the acceptability of different feedstuffs varies according to their characteristics (e.g. whether or not they contain animal by-products, or their legality). The variation between different feeds, respondents, and feed:job combinations is shown below the dashed line. Model weights are proportional to the size of the points. Error bars are 89% credible intervals.

Respondents thought that feeds containing animal by-products or which had the potential for intra-species recycling were less acceptable than feeds which did not (Fig 1)–equivalent to a 1.0-point and 0.7-point lower acceptability (scored from 1–5), respectively, than feeds without (Figure D in S2 Appendix). While the difference between the acceptability of legally permitted and non-legally permitted sources of feed was close to zero (Fig 1), a model including an interaction between job and legal status (model AC1) had similar Akaike weight to models not including this interaction (Table 2), so while it appears that agricultural stakeholders thought that legal feeds and the non-legal feeds were equally acceptable, we cannot rule out that pig farmers perceived feeds that are not legally permitted to be less acceptable than feeds that are currently permitted. Pig farmers, for example, were more accepting of using unsold bread from supermarkets as feed than other respondents were (mean acceptability of 4.26 vs 4.04, Figure C in S2 Appendix), while other respondents were more accepting of the use of heat-treated restaurant left-overs (mean acceptability of 3.17 vs 2.69). There was however, far greater variability between respondents than between the scores for different feeds (compare estimates of the variation between feeds and respondents, the data below the dashed line in Fig 1), indicating no consensus in the acceptability of different food losses as feed.

### Comparison of swill and conventional grain- and soy-based feed

**Fig 2:**
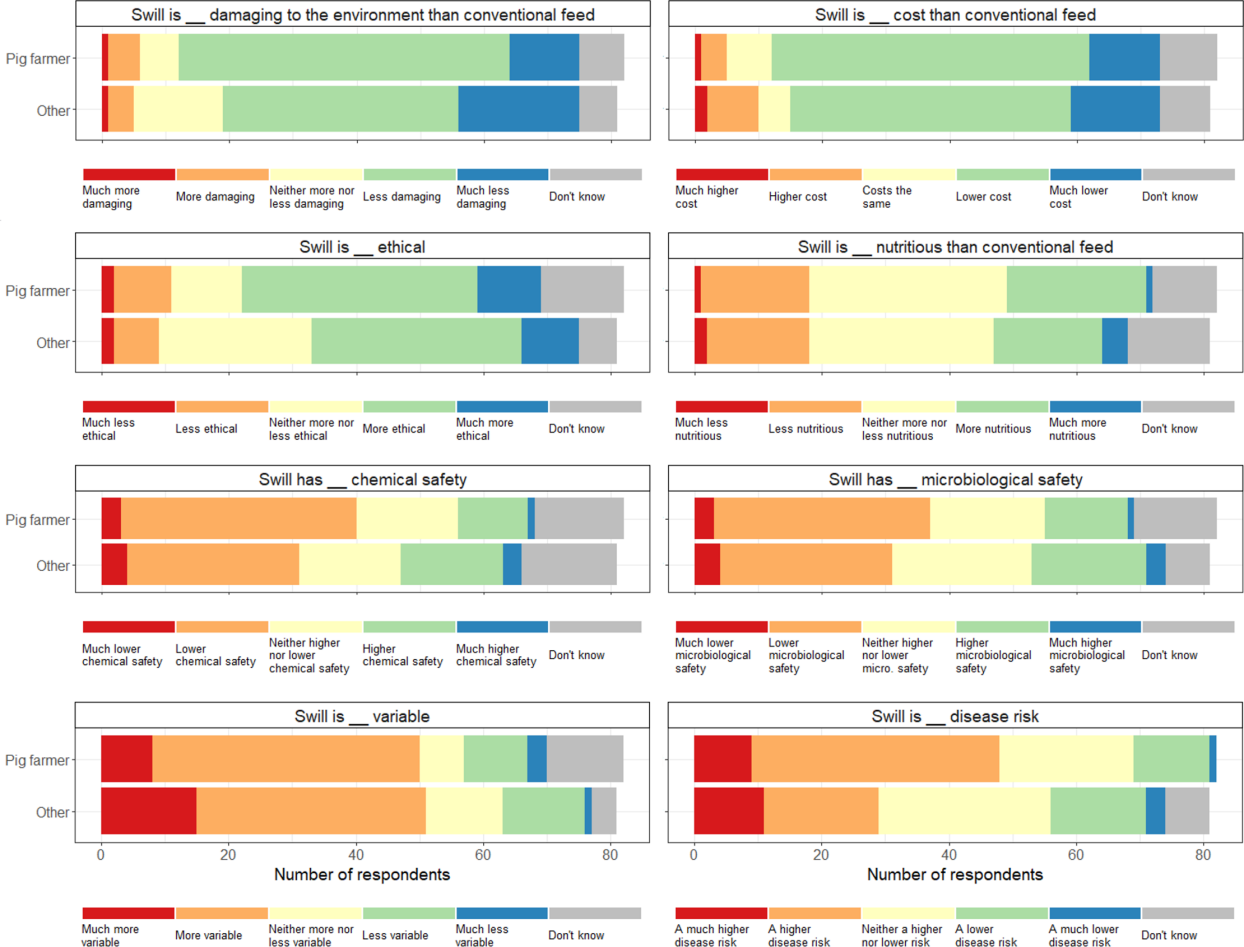
Comparison of swill and conventional feed. Responses to the question: “Compared with feeding conventional grain- and soybean-based feed, heat-treated swill is:”.

Most respondents thought that heat-treated swill is better for the environment, lower cost, and more ethical than conventional feeds, but more variable in nutritional content, and associated with a higher disease risk, lower microbiological safety, and lower chemical safety (Fig 2). There was split opinion about whether swill is more nutritious than conventional feeds, with 22% of respondents thinking that swill was less nutritious, 27% thinking it was more nutritious, and 37% responding “neither more nor less” (with 14% “don’t know”). There was a similar distribution of opinions among both pig farmers and other agricultural stakeholders, except for the question about disease risks, where 58% of pig farmers thought heat-treated swill posed a higher risk (“much higher” or “higher disease risk”), compared with 36% of other respondents.

Respondents thought that using swill would have little effect on animal welfare (75% reported that pigs fed swill would have neither higher nor lower welfare) and would lower feed costs for farmers (84% reported swill would lead to “lower” or “much lower” feed costs; Figure E in S2 Appendix). Respondents were, however, unsure about the impact of swill-feeding on pig performance and meat quality (Figure E and Figure F in S2 Appendix). Twenty-five percent and 28%, respectively, of respondents replied “don’t know” to questions about the effect of swill on pig growth rates and their feed conversion (i.e. how many kilograms of feed are required per kilogram of growth). Similarly, there was uncertainty about the effect of swill-feeding on pork colour (39% of respondents thought there would be no effect, 44% don’t know), taste (46% no effect, 28% don’t know), smell (55% no effect, 29% don’t know), and fattiness (55% no effect, 29% don’t know).

### Opinion on the relegalisation of swill

**Fig 3:**
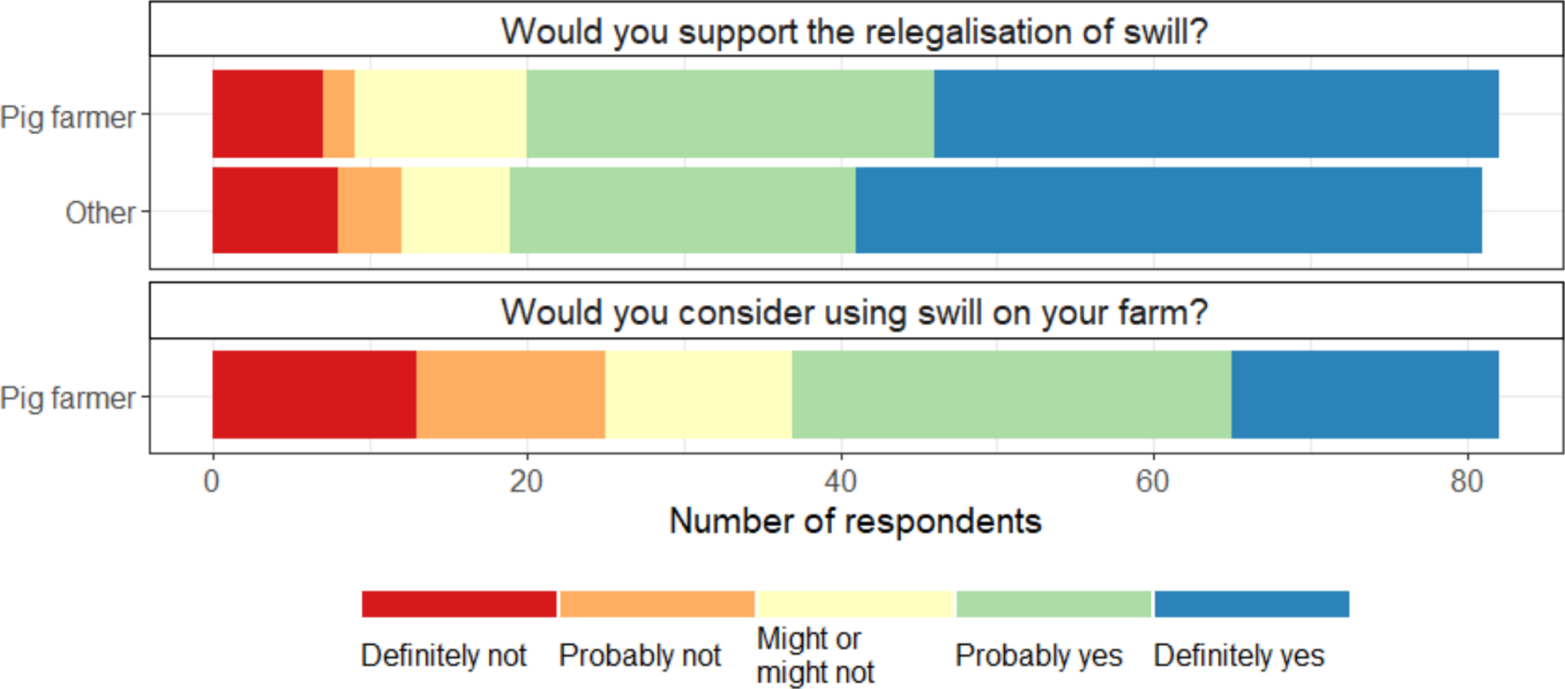
Support for the relegalisation of swill amongst pig farmers and other agricultural stakeholders. Response to the question: “If the procedures were put in place to ensure the safety of swill (e.g. heat treatment was performed by regulated swill manufacturers), would you support the relegalisation of swill?”.

If procedures were put into place to ensure swill was heat-treated, support for its relegalisation was high: 76% and 77%, respectively, of pig farmers and other respondents said they would probably or definitely support the relegalisation of swill (Fig 3); though some were strongly opposed: in total, nine percent of respondents would definitely not support its relegalisation (Fig 3). Most respondents (82%) considered using swill as a traditional farming practice, and 17% thought that it was unnatural (Figure G in S2 Appendix).

**Fig 4:**
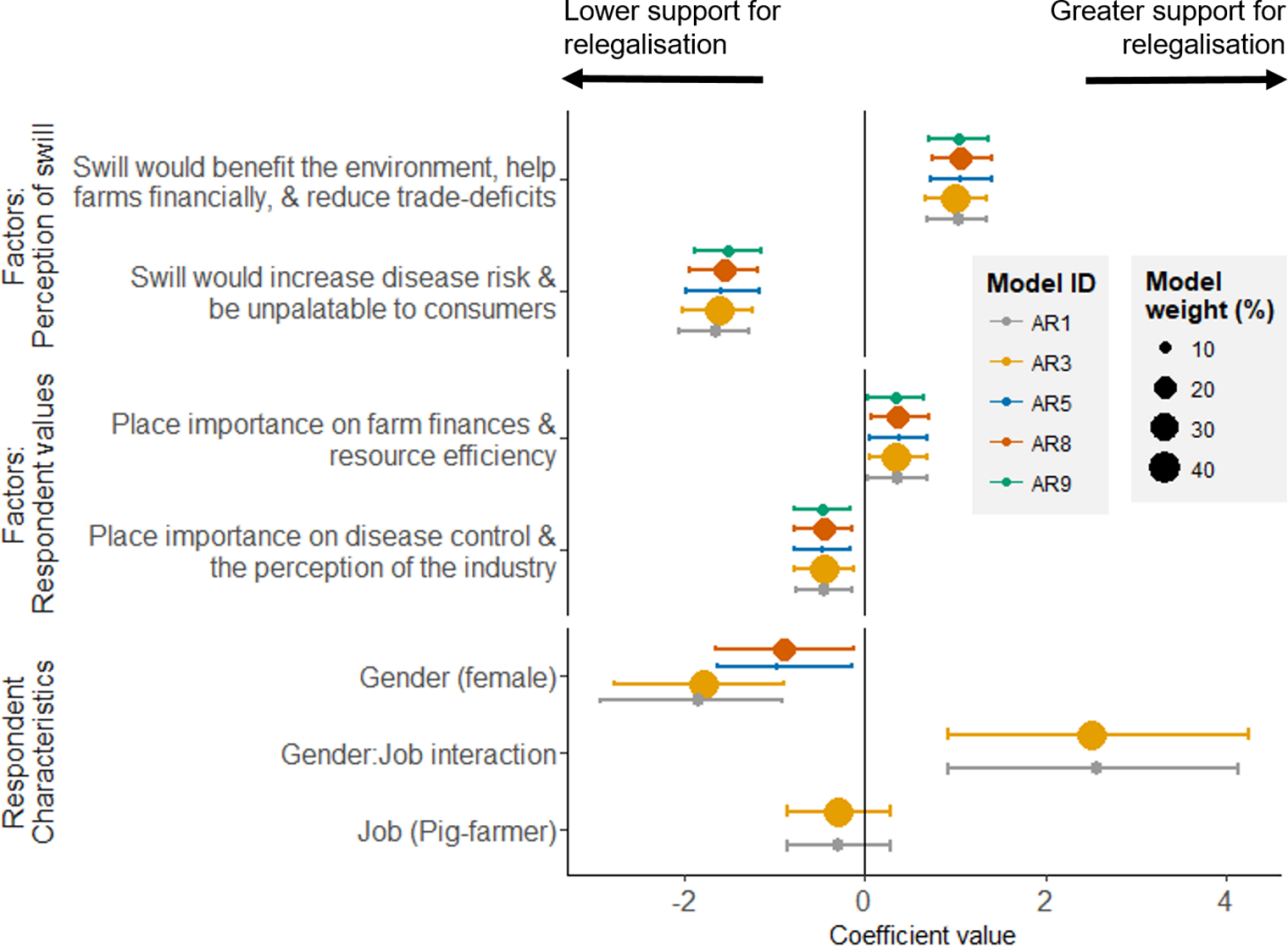
Predictors of the support for the relegalisation of swill, among all respondents (n = 163). The estimates plotted are from the five models with the greatest weighting (85% of model weight), where different colours are used for each model (listed in Table 3) and model weights are proportional to the size of the points. Error bars are 89% credible intervals. For clarity, the coefficients for age groups, which was included in two models, are not plotted here; these are shown in Figure J in S2 Appendix.

Respondent’s opinions on relegalisation were better predicted by their values and perceptions of swill than their characteristics (e.g. age, job, gender). Respondents for whom disease control and the perception of the pork industry by consumers were important (i.e. respondents who scored highly on the factor 1 from the factor analysis about farmer values), supported relegalisation less, while people who were more concerned with financial performance and farm efficiency (factor 2 about farmer values) were more supportive of relegalisation (Fig 4).

People who thought that swill would benefit the environment, help farms financially, and reduce trade-deficits (factor 1 from the factor analysis about the perceived impacts of swill) were more supportive, and people who thought that swill would increase disease risks and be unpalatable to consumers (factor 2 for the perceived impacts of swill) were less supportive (Fig 4).

**Fig 5:**
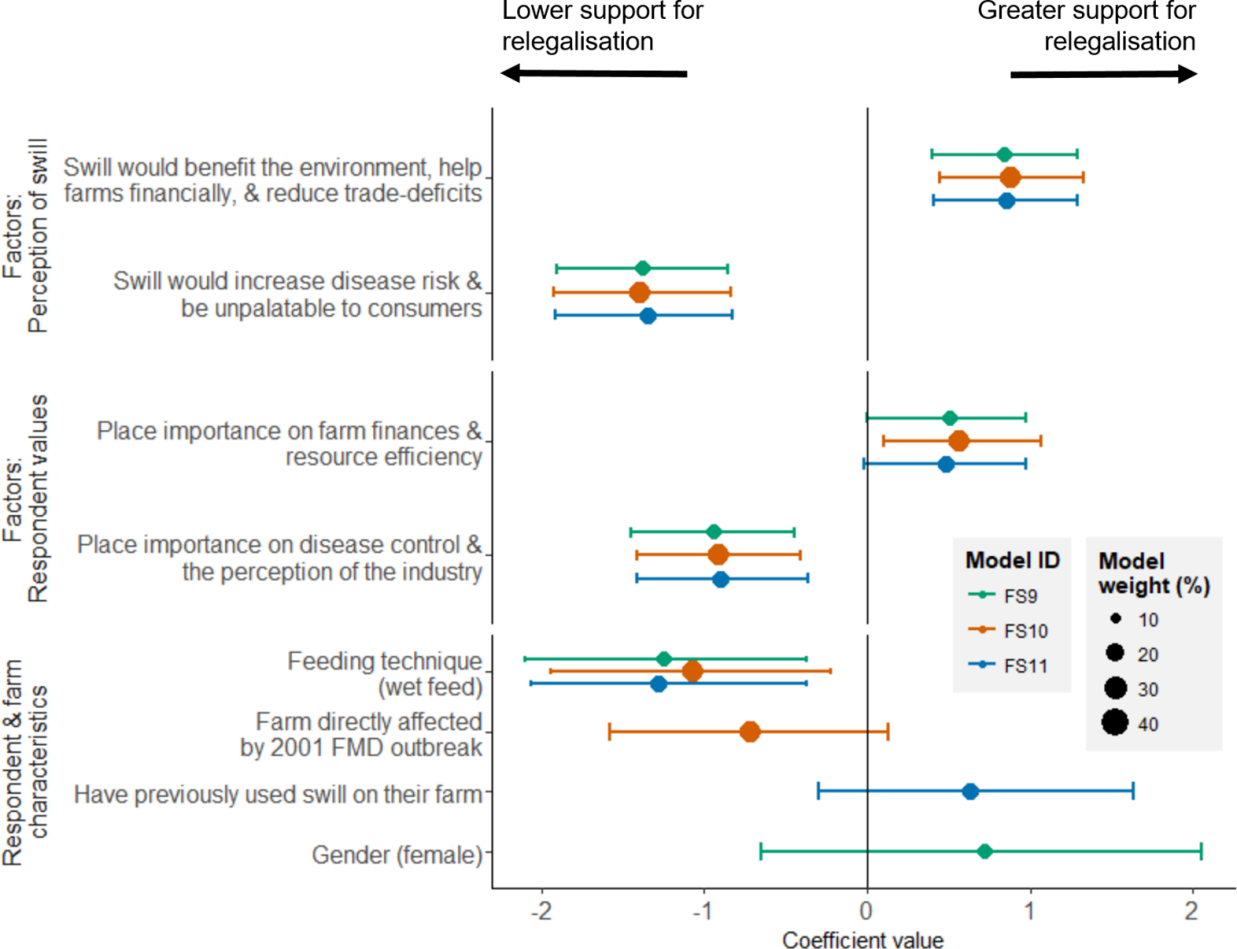
Predictors of farmer support for the relegalisation of swill (n = 82). The top three models shown had 55% of the model weight, and the structure of all models are listed in Table A in S2 Appendix. Error bars are 89% confidence intervals.

There was little difference in support between age-groups (i.e. the difference between age groups was close to zero, Figure J in S2 Appendix). While the model including an interaction between gender and job had the lowest WAIC (Table 3), suggesting that female pig farmers were more supportive of relegalisation, the importance of gender and job in predicting support for the relegalisation of swill should be treated with caution. First, our data included only a small sample of female respondents (21/163 respondents). Second, we suspect that the gender difference in the highest weighted model may be partly explained by other, latent variables. In our sample, 1/7 female farmers were affected by the 2001 foot-and-mouth outbreak (14%), while 21/74 male farmers were affected (28%), which may in part explain why female farmers had higher acceptance for swill-based feeds. Farmers who were affected by the 2001 foot-and-mouth outbreak were less likely to support the relegalisation of swill (Fig 5). Finally, the measured difference between jobs was close to zero (Fig 4), suggesting similar levels of support for the relegalisation of swill among pig-farmers and other agricultural stakeholders (Fig 3).

Amongst pig farmers, there were no differences in support for relegalisation between farm sizes or age groups (models including these parameters had low model weights; Table B in S2 Appendix). Similar to the model fit to all respondents, farmer’s values and perceived impacts of swill were also important predictors of support for relegalisation (Fig 5). Farmers who used wet feeding were less likely to support relegalisation (Fig 5, equivalent to a 0.37 lower score for support for relegalisation; Figure K in S2 Appendix). Models including whether farmers had previous experience using swill (which increased support for relegalisation), gender (female respondents showed greater support for relegalisation), and whether farms were directly affected by the 2001 foot-and-mouth outbreak (lower support if the farm was directly affected) were weighted almost equally, suggesting these may have played a role in farmer willingness to support swill, but their relative importance is uncertain.

**Fig 6:**
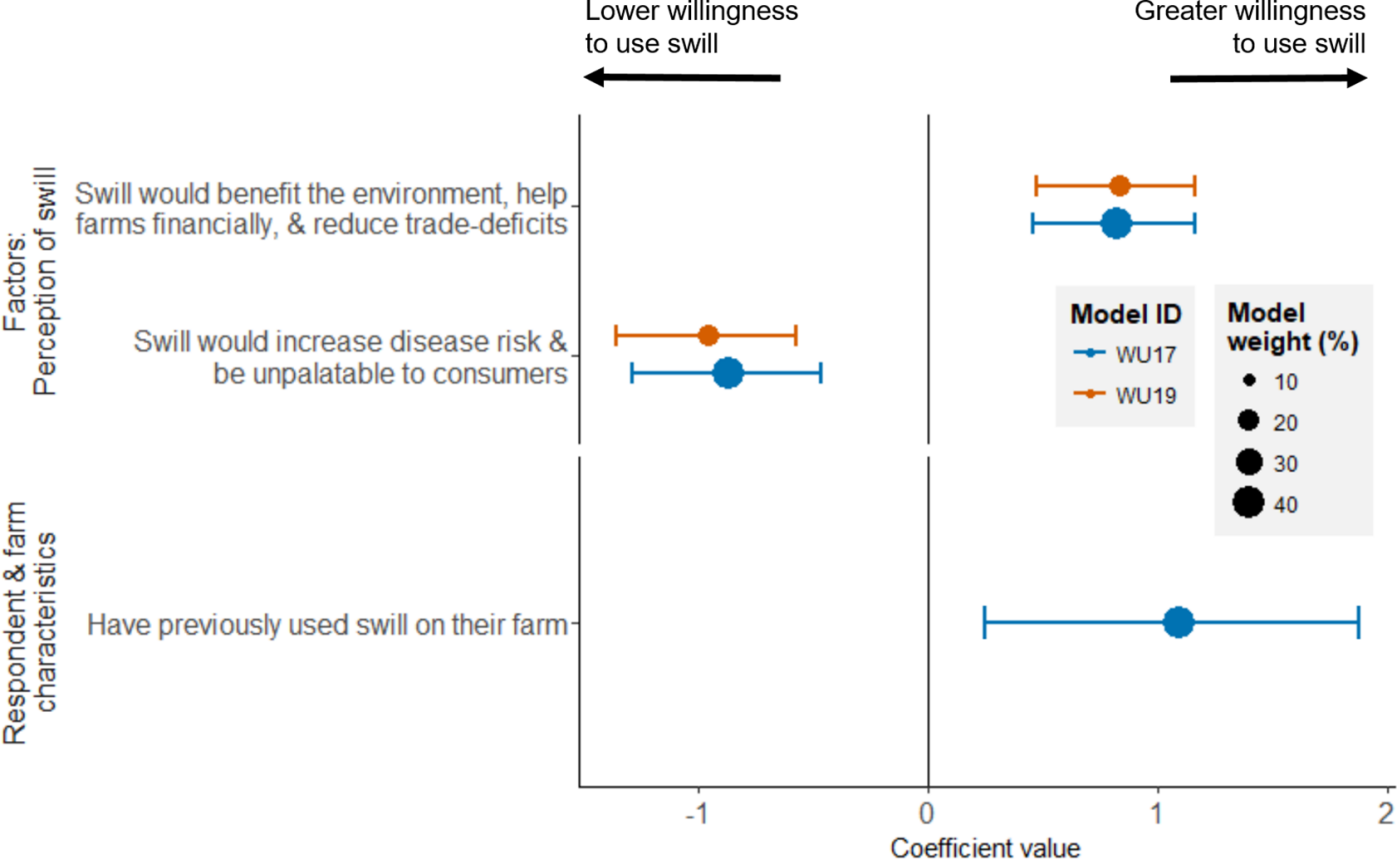
Predictors of farmer willingness to use swill, if it were relegalised (n = 82). The top two models shown had 60% of the model weight; all models are listed in Table B in S2 Appendix.

A lower proportion of respondents (55%) were willing to use swill on their farm if it were relegalised than supported relegalisation per se (Fig 3). In models exploring willingness to use swill on their farm, the perceived impact of swill was more important than the farmer’s values (Fig 6 and Table B in S2 Appendix). Unlike the model predicting support for relegalisation, the farmer’s experience of foot-and-mouth disease, feeding technique, or gender did not affect their willingness to use swill. According to the model which had the greatest weight (Table B in S2 Appendix), farmers who had previously used swill had a 0.6-point higher willingness to use swill, if it were relegalised (Figure L in S2 Appendix).

## Discussion

While respondents thought that feeds containing animal by-products and with a potential for intra-species recycling were less acceptable than those without, and pig farmers showed a preference for feeds that are currently legally permitted, this did not translate into support for the current ban. Overall, we found high support (>75%) for the relegalisation of swill, among both pig farmers and other agricultural stakeholders. Support for swill feeding arose in part because respondents thought that the relegalisation of swill would lower costs, increase profitability, and be better for the environment (Figs 2 and 4), perspectives supported by previous literature on the economics and environmental impacts of using food losses as feed [7,11,24,25].

Though fewer farmers were willing to use swill on their farm than would support its relegalisation, more than half of all farmers reported that they would consider using swill, if it were relegalised (Fig 3). While farmer values predicted their support for relegalisation, they did not predict their willingness to use swill on farm (Fig 6), suggesting, perhaps unsurprisingly, that the business decision about which feeds to use are based more on the practicalities of using a particular feed than less tangible “values”.

Our results also confirm the critical importance of disease control and consumer communication when considering the relegalisation of swill. Many respondents were concerned that using swill could increase the risk of a disease outbreak, and lower the chemical and microbiological safety of the feed (Fig 2)–and these concerns influenced their position on relegalisation. Respondents who thought that swill would increase disease risks and be unpalatable to consumers were less supportive of relegalisation (Fig 4). Perhaps reassuringly, a survey of 1500 consumers in Japan found that they did not perceive Ecofeed-fed pork (Ecofeed is the Japanese name for feed derived from food losses), differently from conventional pork [26], and we also found a strong consensus that respondent’s thought of swill was a traditional, and not an unnatural, feed–contrary to the fears of some UK supermarkets [13].

Farmers’ experience was also important in determining their position on relegalisation. Farmers who were directly affected by the foot-and-mouth outbreak in 2001 (caused by the illegal feeding of uncooked swill) were less likely to support relegalisation, and farmers who had experience using swill in the past were more supportive. While we found that women were, on average, more supportive of the use of swill, we caution against placing a lot of weight on this finding, given the small sample size.

Contrary to our expectation, farmers who used wet feeding were less supportive of relegalisation. As swill has traditionally been fed as a wet feed, these farmers would, in theory be better placed to use many sources of swill, if it were relegalised. Since swill is also fed as a dehydrated pellet in the modern systems of swill feeding in East Asia [27], and wet/dry feeding did not predict actual willingness to use swill, further research is needed to identify the underlying cause for the difference in support for relegalisation between these two groups.

### Study limitations

Our sample size is not trivial–we sampled 82 pig farmers, including 60 who have farms larger than 1,000 animals, making up approximately 4% of the 1,410 large pig farms in the UK [7]. Our results therefore indicate that there is support for the relegalisation of swill among even large-scale producers–a group that previous work has suggested would be less supportive than smallholder pig farmers [2]. As with all voluntary surveys, there is, however, the possibility of sample selection bias, where people with strong opinions on the topic chose to complete the survey, while people with less strong opinions are under-sampled. From our observation of the two days of the fair, we believe that this concern is not the case here. Many respondents completed the survey at the stand and appeared to be as much motivated by the opportunity to sit and sample complimentary food and drink, and enter a prize draw, as by the specific topic of the survey. Similarly, our sample was collected at a trade-fair and it would be interesting to see how our results would differ if collected in different settings–e.g. at smallholder conventions, internet forums, or ideally, through truly random sampling.

While our sample is representative of the attendees of the UK’s largest pig and poultry trade fair, it is also not clear how generalisable our results are to other countries. As the UK was the hardest hit by the 2001 foot-and-mouth outbreak, and other European countries had well developed swill feeding industries, prior to the ban on the use of swill [2], it is possible the UK agriculture sector may be more sceptical of the relegalisation of swill than in other countries. Future work should also evaluate support for the use of swill among other groups, such as the retail sector or the general public.

It is worth noting, however, that the high level of support for swill that we observed is in line with previous surveys on the acceptability of novel animal feeds. Verbeke et al. surveyed 415 farmers and other stakeholders attending a trade-fair in Belgium, finding that 78% of people were supportive of the use of insects in animal feed [16]. Similarly, Gachango et al. surveyed 72 fish farmers in northern Europe (the UK, Germany, Denmark, and Norway), finding that 84% were willing to use pig by-products (bristles and hooves) in fish feed [28]. These results suggest that livestock farmers are not averse to the inclusion of animal by-products in the feed mix of omnivorous species. While swill is still banned in the EU, the ban on the use of monogastric processed animal proteins (such as pig bristles and hooves) in fish feed was lifted in 2013 [29], the ban on the inclusion of insects in fish feed was lifted in 2017 [30], and the relegalisation of insects in poultry feed is expected in 2019 [31].

Finally, our questionnaire asked whether respondents would support the relegalisation of swill, *if* practices for its safe inclusion in feed were introduced. Of course, questions remain about how the technologies and system for recycling food losses operating in East Asia could be best adapted to suit the UK or European context. A UK government risk assessment, for example, concluded that heat-treatment is sufficient to render food losses containing animal by-products safe for animal feed–the risk for the introduction of diseases comes, however, not from the failure of the heat treatment process itself, but from contamination of feed with material that evaded heat treatment [32]. Any new system for the use of swill will therefore require careful design of regulation and operating procedures to reduce the risk of uncooked animal by-products entering feed to a negligible level. Our results suggest, however, that if such a system for safe swill feeding can be established, there would be widespread support amongst UK pig farmers and other agricultural stakeholders for its relegalisation.

## Supporting information

S1 Appendix Copy of survey used in this study. (DOCX)

S2 Appendix Model equations and additional results. (DOCX)
