# Supplementary material for: Support amongst UK pig farmers and agricultural stakeholders for the use of food losses in animal feed
Source: PLoS One. 2018 Apr 24;13(4):e0196288. doi: 10.1371/journal.pone.0196288 (PMC5916861; doi:10.1371/journal.pone.0196288)
Supplement: S1 Appendix — (DOCX) [file pone.0196288.s001.docx]

Please complete this survey about the use of swill as pig feed, contribute to University research, and don’t miss out on the opportunity to **win one of FIVE £50 cash prizes.**

You do not need to be a pig farmer to participate.

The survey should take less than 15 minutes to complete.

Please return your completed survey to stall 359A or leave it with one of our team of researchers (wearing maroon t-shirts) who will be collecting them at the exit to the Blackdown buildings.

**Thank you for your contribution!**

**The reason for the survey:**

While the use of swill (food leftovers) as animal feed is currently banned in the EU, there are some calls for its re-introduction, following the example of countries like Japan and South Korea which operate regulated systems for heat-treating their food leftovers and recycle 40% of food leftovers as feed.

Our survey wants to hear your opinion on the use of swill as pig feed.

1. **Compared with feeding conventional grain- and soybean-based feed, heat-treated swill is:**

| Much less damaging to the environment | Less damaging to the environment | Neither more nor less damaging to the environment | More damaging to the environment | Much more damaging to the environment | Don’t know |
| --- | --- | --- | --- | --- | --- |
| 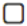 | 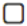 | 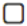 | 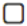 | 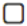 | 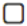 |

| Much less nutritious | Less nutritious | Neither more nor less nutritious | More nutritious | Much more nutritious | Don’t know |
| --- | --- | --- | --- | --- | --- |
| 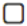 | 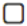 | 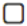 | 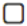 | 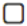 | 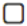 |

| Much less variable in nutritional content | Less variable in nutritional content | Neither more nor less variable in nutritional content | More variable in nutritional content | Much more variable in nutritional content | Don’t know |
| --- | --- | --- | --- | --- | --- |
| 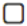 | 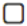 | 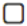 | 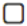 | 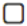 | 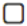 |

| Much lower cost | Lower cost | Costs the same | Higher cost | Much higher cost | Don’t know |
| --- | --- | --- | --- | --- | --- |
| 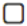 | 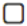 | 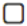 | 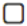 | 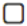 | 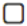 |

| A much lower disease risk | A lower disease risk | Neither higher nor lower disease risk | A higher disease risk | A much higher disease risk | Don’t know |
| --- | --- | --- | --- | --- | --- |
| 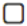 | 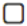 | 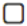 | 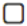 | 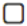 | 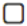 |

| Has much lower microbiological safety | Has lower microbiological safety | Has neither higher nor lower microbiological safety | Has higher microbiological safety | Has much higher microbiological safety | Don’t know |
| --- | --- | --- | --- | --- | --- |
| 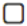 | 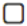 | 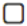 | 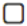 | 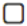 | 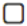 |

| Has much lower chemical safety | Has lower chemical safety | Has neither higher nor lower chemical safety | Has higher chemical safety | Has higher chemical safety | Don’t know |
| --- | --- | --- | --- | --- | --- |
| 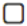 | 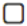 | 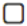 | 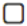 | 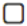 | 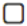 |

| Much less ethical | Less ethical | Neither more nor less ethical | More ethical | Much more ethical | Don’t know |
| --- | --- | --- | --- | --- | --- |
| 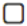 | 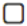 | 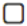 | 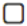 | 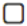 | 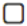 |

1. **How would you feel about the inclusion of the following in pig feed:**

*Please tick which box applies to each row.*

|  | Very negative | Negative | Neither positive nor negative | Positive | Very positive |
| --- | --- | --- | --- | --- | --- |
| Heat-treated household food leftovers | 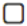 | 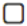 | 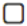 | 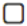 | 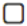 |
| Misshapen chocolates from chocolate factories | 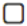 | 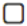 | 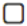 | 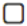 | 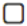 |
| Heat-treated, unsold chicken sandwiches from supermarkets | 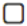 | 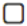 | 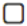 | 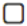 | 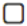 |
| Heat-treated leftovers from a college canteen | 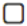 | 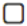 | 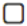 | 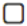 | 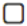 |
| Unsold bread from supermarkets | 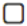 | 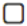 | 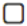 | 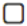 | 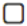 |
| Unsold egg sandwiches from supermarkets | 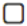 | 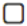 | 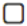 | 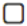 | 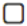 |
| Unsold confectionary containing porcine gelatine | 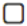 | 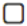 | 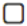 | 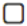 | 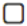 |
| Biscuit crumbs from biscuit factories | 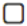 | 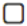 | 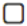 | 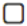 | 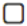 |
| Heat-treated, unsold bacon sandwiches from supermarkets | 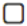 | 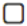 | 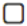 | 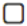 | 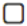 |
| Heat-treated restaurant leftovers | 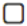 | 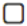 | 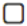 | 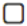 | 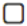 |

1. **How would you feel about the inclusion of the following in pig feed:**

*Please tick which box applies to each row.*

|  | Very uncomfortable | Uncomfortable | Neither comfortable nor uncomfortable | Comfortable | Very Comfortable |
| --- | --- | --- | --- | --- | --- |
| Heat-treated, unsold chicken sandwiches from supermarkets | 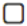 | 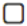 |  |  |  |
| Unsold confectionary containing porcine gelatine |  |  |  |  |  |
| Heat-treated, unsold bacon sandwiches from supermarkets |  |  |  |  |  |
| Biscuit crumbs from biscuit factories |  |  |  |  |  |
| Heat-treated household food leftovers |  |  |  |  |  |
| Heat-treated restaurant leftovers |  |  |  |  |  |
| Misshapen chocolates from chocolate factories |  |  |  |  |  |
| Unsold bread from supermarkets |  |  |  |  |  |
| Unsold egg sandwiches from supermarkets |  |  |  |  |  |
| Heat-treated leftovers from a college canteen |  |  |  |  |  |

1. **How would you feel about the inclusion of the following in pig feed:**

*Please tick which box applies to each row.*

|  | Very dissatisfied | dissatisfied | Neither satisfied nor dissatisfied | Satisfied | Very satisfied |
| --- | --- | --- | --- | --- | --- |
| Heat-treated restaurant leftovers |  |  |  |  |  |
| Biscuit crumbs from biscuit factories |  |  |  |  |  |
| Unsold confectionary containing porcine gelatine |  |  |  |  |  |
| Unsold bread from supermarkets |  |  |  |  |  |
| Unsold egg sandwiches from supermarkets |  |  |  |  |  |
| Heat-treated leftovers from a college canteen |  |  |  |  |  |
| Misshapen chocolates from chocolate factories |  |  |  |  |  |
| Heat-treated, unsold bacon sandwiches from supermarkets |  |  |  |  |  |
| Heat-treated, unsold chicken sandwiches from supermarkets |  |  |  |  |  |
| Heat-treated household food leftovers |  |  |  |  |  |

1. **To what extent do you agree with the following statements?**

**Using heat-treated swill would…**

|  | Totally disagree | Disagree | Neither agree nor disagree | Agree | Totally agree | Don’t know |
| --- | --- | --- | --- | --- | --- | --- |
| Lower dependence on foreign protein sources |  |  |  |  |  |  |
| Reduce the environmental impact of food waste disposal |  |  |  |  |  |  |
| Reduce the environmental impact of pork production |  |  |  |  |  |  |
| Help farms reduce feed costs |  |  |  |  |  |  |
| Help farmers improve profitability |  |  |  |  |  |  |
| Lower consumer acceptance of pork products |  |  |  |  |  |  |
| Increase the risk of an outbreak of foot-and-mouth disease |  |  |  |  |  |  |
| Increase the risk of prion diseases like BSE (mad cow disease) or vCJD (Creutzfeldt-Jacob disease) |  |  |  |  |  |  |
| Increase the risk of toxins entering the feed |  |  |  |  |  |  |
| Reduce the traceability of feed production |  |  |  |  |  |  |
| Be an efficient way to use food waste |  |  |  |  |  |  |
| Negatively affect the marketability of pork |  |  |  |  |  |  |

1. **Compared with pigs fed conventional diets, pigs fed heat-treated swill have:**

| Much slower growth rates | Slower growth rates | Neither faster nor slower growth rates | Faster growth rates | Much faster growth rates | Don’t know |
| --- | --- | --- | --- | --- | --- |
|  |  |  |  |  |  |

| \|  \| \| **Much higher feed conversion ratios (less efficient)** \| \| --- \| \| \| --- \| --- \| --- \| | Higher feed conversion ratios (less efficient) | Has neither higher nor lower feed conversion ratios | Lower feed conversion ratios (more efficient) | Much lower feed conversion ratios (more efficient) | Don’t know |
| --- | --- | --- | --- | --- | --- | --- | --- | --- |
|  |  |  |  |  |  |

| Much lower welfare | Lower welfare | Neither higher nor lower welfare | Higher welfare | Much higher welfare | Don’t know |
| --- | --- | --- | --- | --- | --- |
|  |  |  |  |  |  |

| Much lower feed costs | Lower feed costs | Neither higher nor lower feed costs | Higher feed costs | Much higher feed costs | Don’t know |
| --- | --- | --- | --- | --- | --- |
|  |  |  |  |  |  |

**SURVEY CONTINUED ON NEXT PAGE…**

1. **Compared with PORK from pigs fed conventional diets, PORK from pigs fed diets containing heat-treated swill is:**

| Much worse for the environment | Worse for the environment | Neither better nor worse for the environment | Better for the environment | Much better for the environment | Don’t know |
| --- | --- | --- | --- | --- | --- |
|  |  |  |  |  |  |

| Much less fatty | Less fatty | Neither more nor less fatty | More fatty | Much more fatty | Don’t know |
| --- | --- | --- | --- | --- | --- |
|  |  |  |  |  |  |

| Much lighter in colour | Lighter in colour | Neither lighter nor darker in colour | Darker in colour | Much darker in colour | Don’t know |
| --- | --- | --- | --- | --- | --- |
|  |  |  |  |  |  |

| Much less tasty | Less tasty | Neither more nor less tasty | More tasty | Much more tasty | Don’t know |
| --- | --- | --- | --- | --- | --- |
|  |  |  |  |  |  |

| Much worse smelling | Worse smelling | Neither better nor worse smelling | Better smelling | Much better smelling | Don’t know |
| --- | --- | --- | --- | --- | --- |
|  |  |  |  |  |  |

| Much less marketable | Less marketable | Neither more nor less marketable | More marketable | Much more marketable | Don’t know |
| --- | --- | --- | --- | --- | --- |
|  |  |  |  |  |  |

| Much less profitable | Less profitable | Neither more nor less profitable | More profitable | Much more profitable | Don’t know |
| --- | --- | --- | --- | --- | --- |
|  |  |  |  |  |  |

1. **To what extent do you agree with the following statements?**

|  | Definitely not | No | Not sure | Yes | Absolutely yes |
| --- | --- | --- | --- | --- | --- |
| Feeding swill is a traditional farming practice |  |  |  |  |  |
| Using swill is an unnatural feeding practice. |  |  |  |  |  |

1. **If the procedures were put in place to ensure the safety of swill (e.g. heat treatment was performed by regulated swill manufacturers), would you support the re-legalisation of swill?**

| Definitely not | No | Not sure | Yes | Definitely yes |
| --- | --- | --- | --- | --- |
|  |  |  |  |  |

1. **When considering the re-legalisation of swill, how much importance do you place on the following considerations?**

|  | Not at all important | Not important | Neither important nor unimportant | Important | Very important |
| --- | --- | --- | --- | --- | --- |
| Food safety |  |  |  |  |  |
| Traceability |  |  |  |  |  |
| Profitability |  |  |  |  |  |
| Meat quality |  |  |  |  |  |
| Communication with consumers |  |  |  |  |  |
| Environmental impacts |  |  |  |  |  |
| Labelling of the end product |  |  |  |  |  |
| Consumer acceptance |  |  |  |  |  |
| Disease control |  |  |  |  |  |
| Feed prices |  |  |  |  |  |
| Efficient use of resources |  |  |  |  |  |
| Perception of the pork industry |  |  |  |  |  |

1. **What is your gender?**

| Male |  |
| --- | --- |
| Female |  |

1. **What is your age bracket?**

| 0-18 |  |
| --- | --- |
| 19-30 |  |
| 31-50 |  |
| 51+ |  |

1. **Please select the profession which best describes your job.**

**If you selected that you are a pig farmer, there are 5 more quick questions, below.**

**If you are not a pig farmer – thank you for completing the survey! Please hand it in to one of our research team (wearing maroon t-shirts) at stand 359A or the exits to the Blackdown buildings.**

**To be in with a chance of winning one of our FIVE £50 cash prizes, please list your email address here _________________________________**

| Pig farmer/pig farm manager |  | Retailer |  |  |
| --- | --- | --- | --- | --- |
| Poultry farmer/poultry farm manager |  | **Student** |  |  |
| Farmer/farm manager of both a pig and poultry farm |  | **Veterinarian** |  |  |
| Trader |  | **Food service industry** |  |  |
| Feed processor |  | **Other: involved in the animal industry** |  | Description: |
| Agricultural advisor |  | **Other: not involved in the animal industry** |  | Description: |

1. **How many pigs do you have at any one time?**

| 1-9 |  |
| --- | --- |
| 10-99 |  |
| 100-199 |  |
| 200-399 |  |
| 400-999 |  |
| 1000-4999 |  |
| 5000+ |  |

1. **Do you use wet or dry feeding?**

| Wet |  |
| --- | --- |
| Dry |  |

1. **Have you ever used swill on your farm before?**

| No | Yes | Not sure |
| --- | --- | --- |
|  |  |  |

1. **If the use of swill were legalised, and procedures were put in place to ensure its feed safety, would you consider using swill on your farm?**

| Definitely not | No | Might or might not | Yes | Definitely yes |
| --- | --- | --- | --- | --- |
|  |  |  |  |  |

1. **Was your farm directly affected by the 2001 Foot and Mouth disease outbreak?**

**Thank you very much for completing our survey and sharing your opinions with us! Please hand it in to one of our research team (wearing maroon t-shirts) at stand 359A or the exits to the Blackdown buildings.**

**To be in with a chance of winning one of our FIVE £50 cash prizes, please list your email address here _________________________________**

**If you have any questions about our research, please don’t hesitate to come to our stand, or send us a message at CambridgeSwillSurvey@gmail.com**

| No | Yes | Not sure |
| --- | --- | --- |
|  |  |  |
