# Supplementary material for: Support amongst UK pig farmers and agricultural stakeholders for the use of food losses in animal feed
Source: PLoS One. 2018 Apr 24;13(4):e0196288. doi: 10.1371/journal.pone.0196288 (PMC5916861; doi:10.1371/journal.pone.0196288)
Supplement: S2 Appendix — (DOCX) [file pone.0196288.s002.docx]

Contents

[Data and code 1](#_Toc511553845)

[Characteristics of survey respondents 1](#_Toc511553846)

[Acceptability of different food losses as feed 2](#_Toc511553847)

[Data on the acceptability of different food losses as feed 2](#_Toc511553848)

[Models of acceptability of different food losses as feed 3](#_Toc511553849)

[Farmer perceptions of swill 4](#_Toc511553850)

[Data used in factor analysis 6](#_Toc511553851)

[Farmer values 6](#_Toc511553852)

[Impacts of swill 7](#_Toc511553853)

[Models of respondents’ support for relegalisation of swill 8](#_Toc511553854)

[Models of farmer support for the relegalisation of swill 10](#_Toc511553855)

[Models of farmer willingness to use swill, if it were relegalised 12](#_Toc511553856)

[References cited in S2 Appendix 15](#_Toc511553857)

# Data and code

The data and code used for all analyses can be found at <https://doi.org/10.17863/CAM.21189>.

# Characteristics of survey respondents


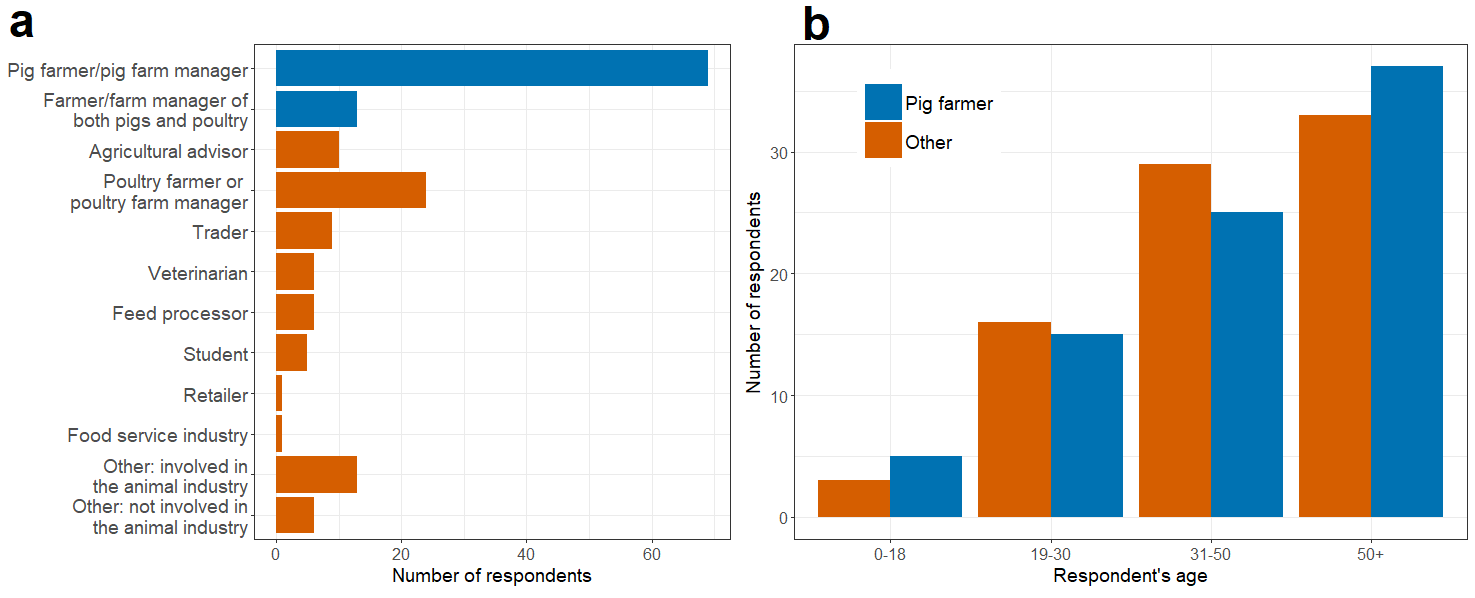


Fig A. Respondent jobs (a) and ages (b).


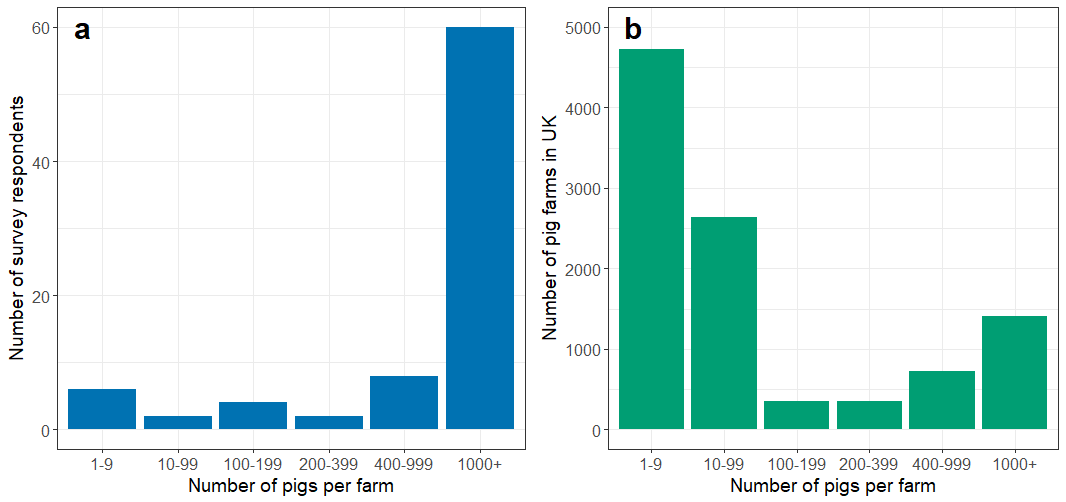


Fig B. (a) Farm size distribution for the 82 pig farmers who completed the survey; (b) farm size distribution for the 10,190 pig farms in the UK. Source: [15].

# Acceptability of different food losses as feed

## Data on the acceptability of different food losses as feed


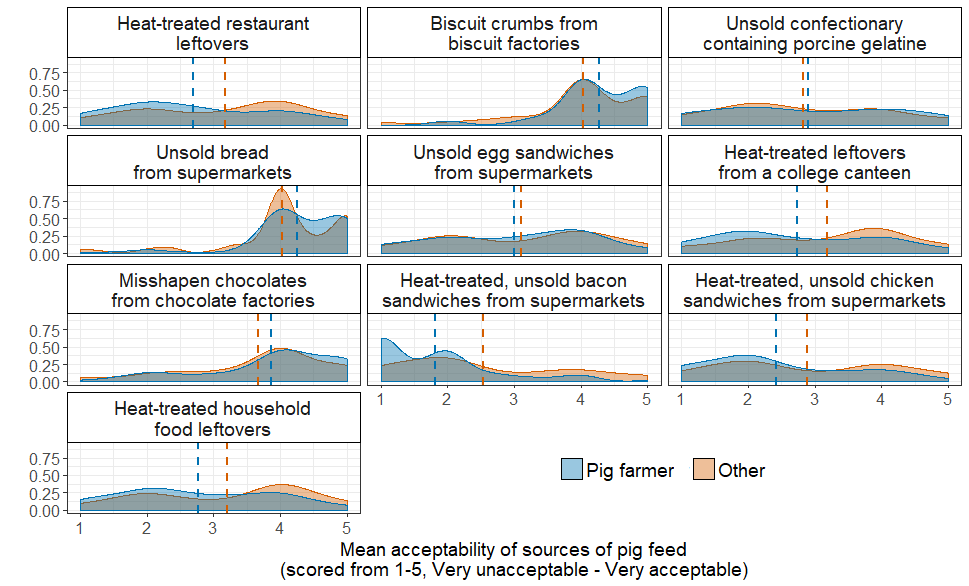


Fig C. Density plot (i.e. a smoothed histogram) of the acceptability of different feeds. Results are the answer to the question: “How would you feel about the inclusion of the following in pig feed?” Respondents were asked to score feeds from 1-5, using three different scales: very uncomfortable – very comfortable, very dissatisfied – very satisfied, very negative – very positive. These different constructs had high internal reliability (alpha = 0.95), and so the mean of these scores per respondent is plotted here. The vertical lines designate the overall mean for each feed, per job group.

## Models of acceptability of different food losses as feed

The structure and priors used for the maximal model, (model AC1) are described in detail below. The predictors included in subsequent models are described in Table 2; these models were fit using the same priors.

**Model AC1:**

**Model structure**

$LikertScore \sim Ordered\left( \varphi\right)$ [likelihood]

$logit\left( \varphi_{k} \right)=\alpha_{k}+ \alpha_{RESPONDENT\left[ i \right]}+ \alpha_{FEED\left[ f \right]}+ \beta_{FEED\left[ f \right]}*J+$ [cumulative link & linear model]

$\beta_{LEGAL}+ \beta_{ABP}+ \beta_{INTRA\_SPP}+ \beta_{JOB,LEGAL}$ […continued]

$\left[ \begin{matrix} \alpha_{FEED} \\ \beta_{FEED} \end{matrix} \right] \sim MVN\left( \left[ \begin{matrix} 0 \\ \beta\end{matrix} \right],S \right)$ [joint distribution for varying effects]

$S= \left( \begin{matrix} \sigma_{\alpha} & 0 \\ 0 & \sigma_{\beta} \end{matrix} \right)R\left( \begin{matrix} \sigma_{\alpha} & 0 \\ 0 & \sigma_{\beta} \end{matrix} \right)$ [covariance matrix]

**Priors**

$\alpha_{k}=Normal(0,10)$ [common prior for each intercept]

$\alpha_{RESPONDENT}=Normal(0,\sigma_{R})$ [prior for respondent intercept]

$(\beta,\beta_{LEGAL}, \beta_{ABP}, \beta_{INTRA_{SPP}})=Normal\left( 0,1 \right)$ [prior for each slope]

${(\sigma}_{\alpha},\sigma_{\beta},\sigma_{R})=HalfCauchy(0,2)$ [prior for each σ]

$R=LKJcorr(4)$ [prior for correlation matrix]

Where,

$LikertScore$ is the score for the acceptability of each feed (1-5, Very unacceptable – Very acceptable, Very negative - Very positive, or Very dissatisfied - Very satisfied).

$Ordered$ is an ordered categorical log-odds probability density function.

$\varphi_{k}$ is the probability of responding in each category *k* (below the maximum category *k+1*).

$\alpha_{k}$ are estimated intercepts for each response category *k.*

$\alpha_{RESPONDENT\left[ i \right]}$ are estimated intercepts for different respondents.

$\alpha_{FEED\left[ f \right]}$ are estimated intercepts for different feed types.

$\beta_{FEED\left[ f \right]}$ are estimated slopes of the interaction between each feed and job.

$J$is the value for job (1=pig farmer, 0=other).

$\beta_{LEGAL}$= slope for the legal status of the feed (0=illegal, 1=legal).

$\beta_{ABP}$ = the slope for whether the feed potentially includes animal by-products (0=no ABPs, 1=may contain ABPs).

$\beta_{INTRA\_SPP}$ = slope for whether or not the feed potentially allows intra-species recycling (0=no intra-species recycling, 1=potential for intra-species recycling).

$\beta_{JOB,LEGAL}$is the slope for the interaction between job (pig farmer=1, other=0), and legal status (0=illegal, 1=legal).

$\beta$ is the slope common to all feeds.

S is the covariance matrix for the two-dimensional Gaussian distribution linking the intercepts ($\alpha_{FEED}$) and slopes ($\beta_{FEED}$) of each feed.

R is the correlation matrix.


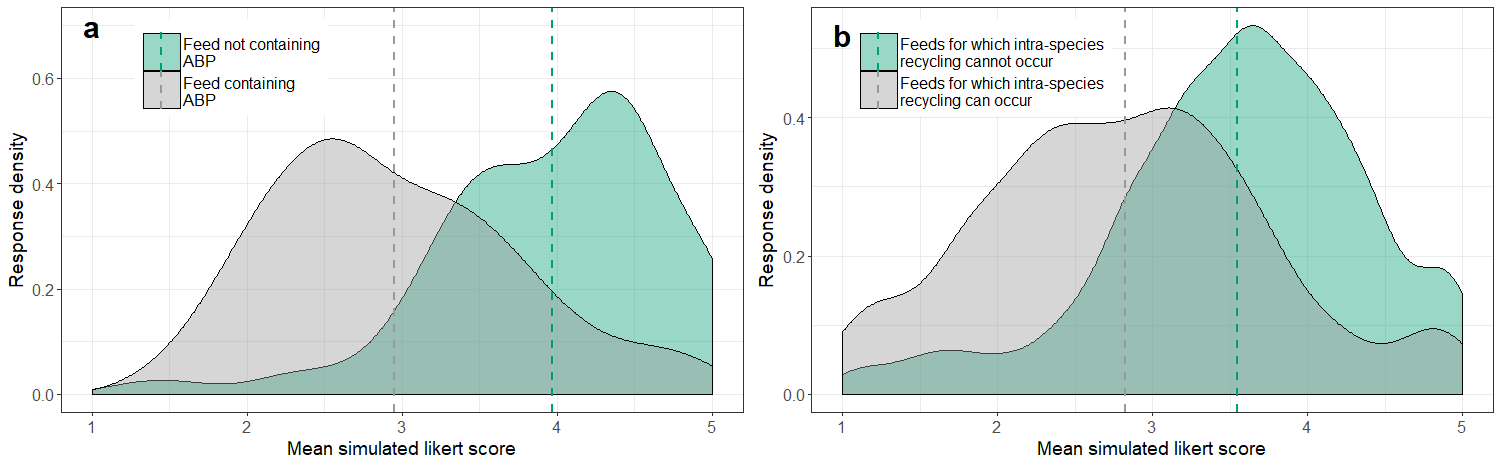


Fig D. Density plot of the acceptability of (a) feeds with and without animal by-products (ABPs), and (b) feeds for which intra-species recycling can/cannot occur. The data are the mean simulated responses from 1000 respondents, based on model averaged output (Table 2). The vertical lines designate the overall mean for each category of feeds.

# Farmer perceptions of swill


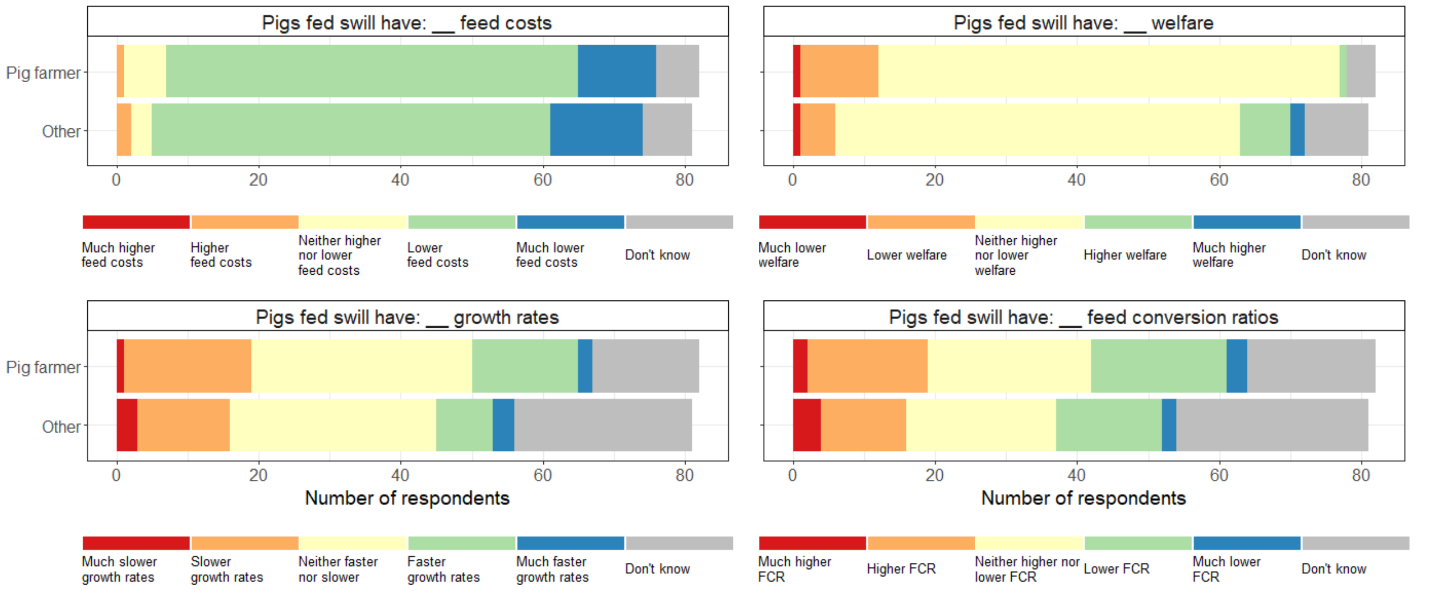


Fig E. Comparison of pig performance when fed swill or conventional diets. FCR = feed conversion ratio (i.e. how many kilograms of feed are required per kilogram of growth). Responses to the question: “Compared with pigs fed conventional diets, pigs fed heat-treated swill have:”.


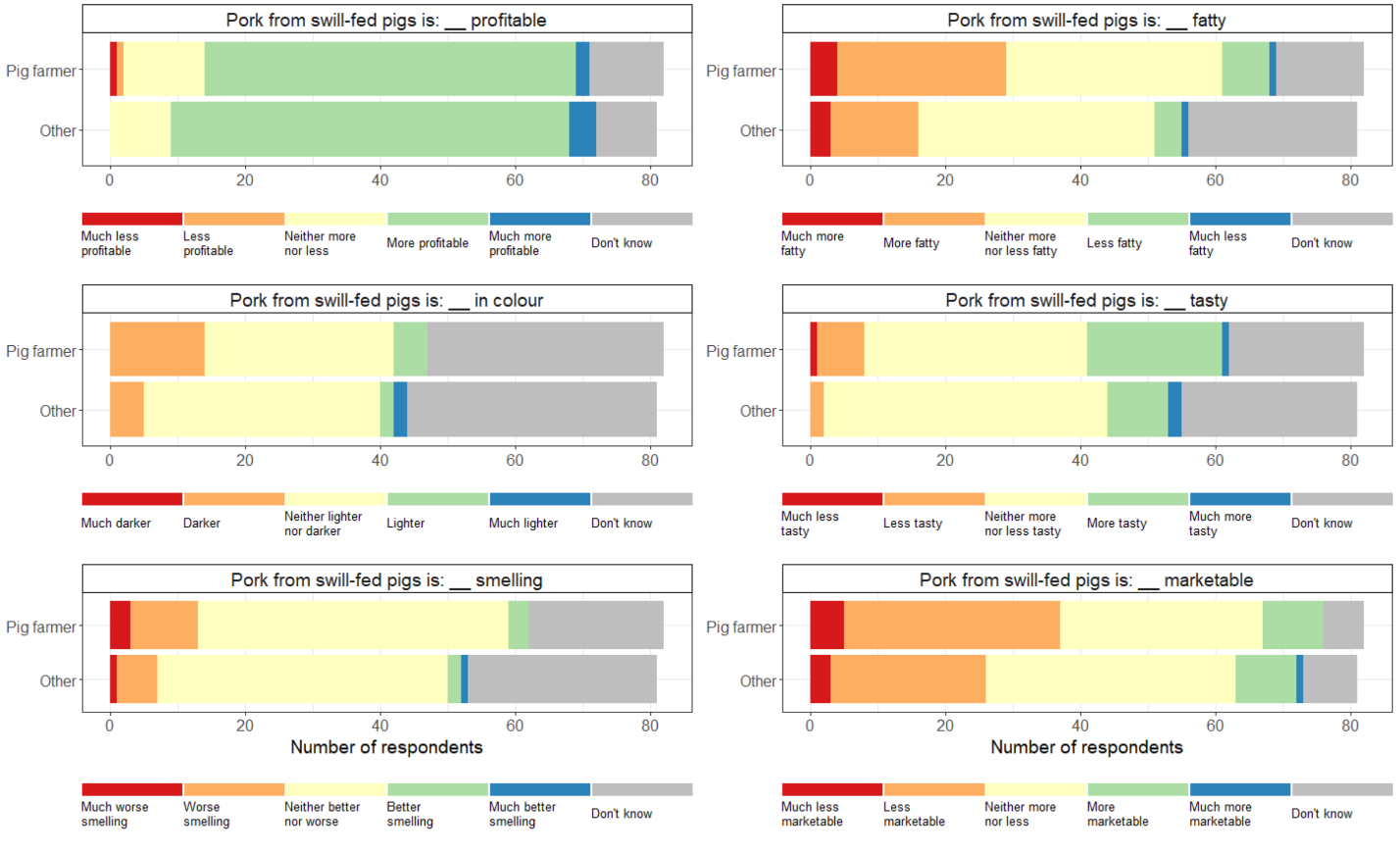


Fig F. Comparison of the attributes of pork from pigs fed swill or conventional feed. Responses to the question: “Compared with pork from pigs fed conventional diets, pork from pigs fed diets containing heat-treated swill is”.


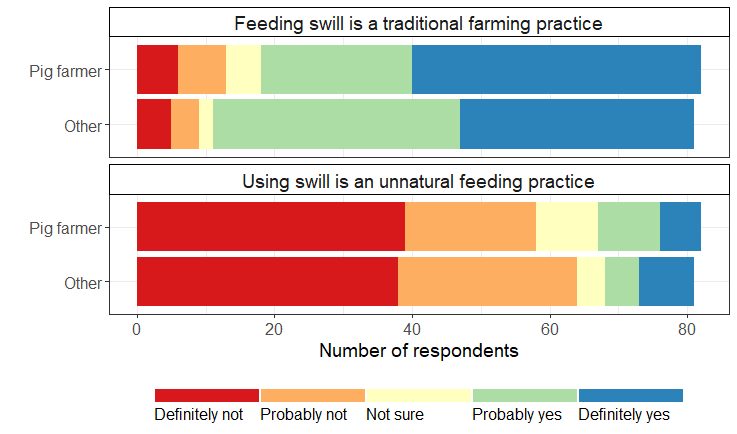


Fig G. The perception of swill as a traditional or an unnatural farming practice. Response to the question: “To what extent do you agree with the following statements?”.

# Data used in factor analysis

## Farmer values

The most important issues for respondents, when considering the relegalisation of swill, were food safety (67% thought it was “very important”, and 30% “important”) and disease control (73% thought it was “very important”, 23% “important”), though all of the identified issues were considered to be important by the majority of respondents (S2 Appendix Fig 8).

These scores for farmer values were simplified using factor analysis, as described in the methods section, and included in models of respondent support for the relegalisation of swill, and farmer willingness to use swill.


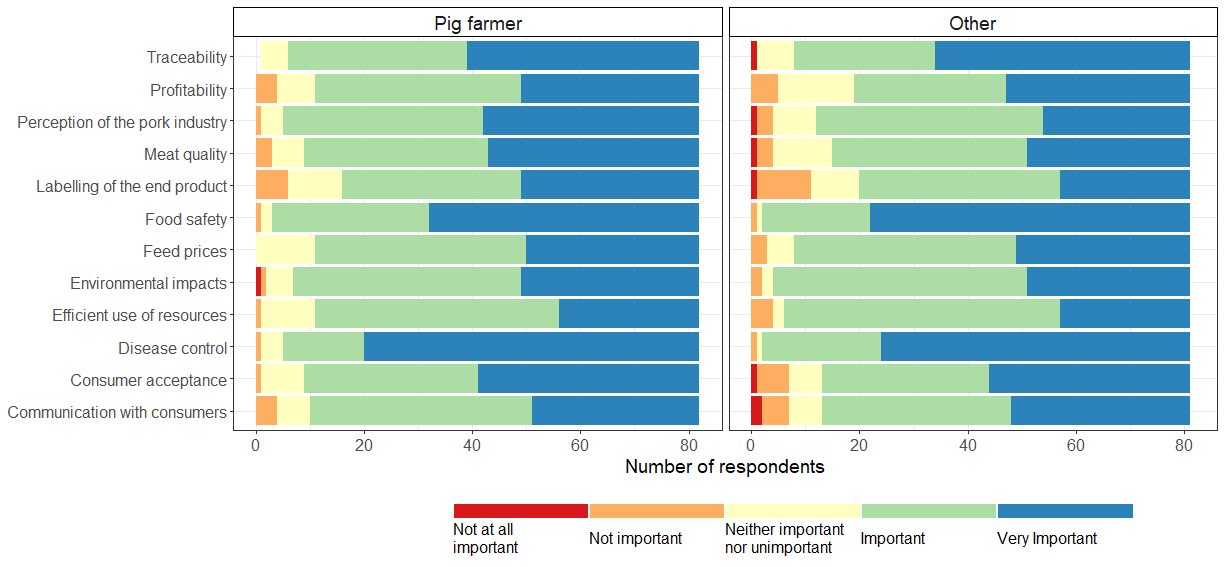


Fig H. The importance of 12 different issues to respondents. Answer to the question: “When considering the relegalisation of swill, how much importance do you place on the following considerations?”. These data were simplified using factor analysis, and included in modelling of the support for relegalisation, and willingness to use swill.

## Impacts of swill

There was very high agreement (>80% “agree” or “totally agree”) that using heat-treated swill would be an efficient way to use food waste, would reduce the environmental impact of pork production, and would lower dependence on foreign protein sources (S2 Appendix Fig 9). Opinions were more evenly split on whether swill would negatively affect the marketability of pork (44% of respondents “agree” or “totally agree”, vs 39% who “disagree” or “totally disagree”), increase the risk of an outbreak of foot-and-mouth disease (35% vs 39%), or lower consumer acceptance of pork (40% vs 33%). It is interesting to note that 40% of respondents thought that using swill would increase the risk of prion diseases (such as BSE, or mad cow disease), though there is no evidence of pigs ever naturally contracting prion diseases [25].

The scores for respondents’ perceptions of the impact of swill were simplified using factor analysis, as described in the methods section, and included in models of respondent support for the relegalisation of swill, and farmer willingness to use swill.


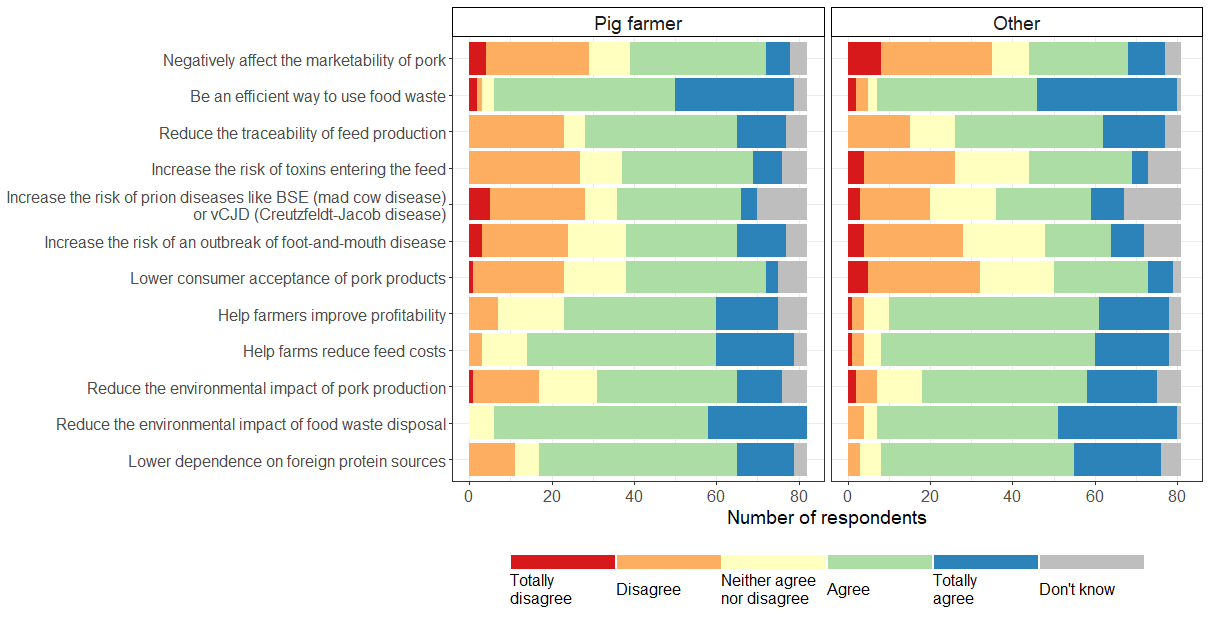


Fig I. Farmer perceptions of the impacts of swill. Response to the question: “Using heat-treated swill would…”. These data were simplified using factor analysis, and included in modelling of the support for relegalisation, and willingness to use swill.

# Models of respondents’ support for relegalisation of swill

The structure and priors used for the maximal model (model AR1) are described below. The predictors included in subsequent models are described in Table 3; these models were fit using the same priors.

**Model AR1:**

**Model structure**

$LikertScore \sim Ordered\left( \varphi\right)$ [likelihood]

$logit\left( \varphi_{k} \right)=\alpha_{k}+ \alpha_{AGE_{GROUP\left[ a \right]}}+\beta_{GENDER}+\beta_{JOB}+\beta_{JOB,GENDER}+$ [cumulative link & linear model]

$\beta_{VAL\_F1}+ \beta_{VAL\_F2}+ \beta_{IMP\_F1}+ \beta_{IMP\_F2}$ […continued]

**Priors**

$\alpha_{k}=Normal(0,10)$ [common prior for each intercept]

$\alpha_{AGE\_GROUP\left[ a \right]}=Normal(0,\sigma_{A})$ [prior for age group intercepts]

$\left( \beta_{GENDER},\beta_{JOB}, {\beta_{JOB,GENDER},\beta}_{VAL_{F1}}, \beta_{VAL_{F2}}, \beta_{IMP_{F1}}, \beta_{IMP_{F2}} \right)=Normal\left( 0,10 \right)$ [Priors for slopes]

$\sigma_{A}=HalfCauchy(0,1)$ [prior for $\sigma_{A}$]

Where,

$LikertScore$ is the score for the support for relegalisation of swill, (1-5, Definitely not – Definitely yes), amongst all respondents (n=163).

$Ordered$ is an ordered categorical log-odds probability density function.

$\varphi_{k}$ is the probability of responding in each category *k* (below the maximum category *k+1*).

$\alpha_{k}$ are estimated intercepts for each response category *k.*

$\alpha_{AGE\_GROUP\left[ a \right]}$ is the intercept for different age groups (shown in S2 Appendix Fig 1).

$\beta_{GENDER}$is the slope for the respondent’s gender (1=female, 0=male).

$\beta_{JOB}$is the slope for the respondent’s job (1=pig farmer, 0=other).

$\beta_{JOB,GENDER}$is the slope for the interaction between job (1=pig farmer, 0=other) and gender (1=female, 0=male).

$\beta_{VAL_{F1}} \& \beta_{VAL_{F2}}$are the slope for the first and second factor loadings for respondent’s values.

$\beta_{IMP\_F1}\& \beta_{IMP\_F2}$ are the slope for the first and second factor loadings for respondent’s perception of the impacts of swill.


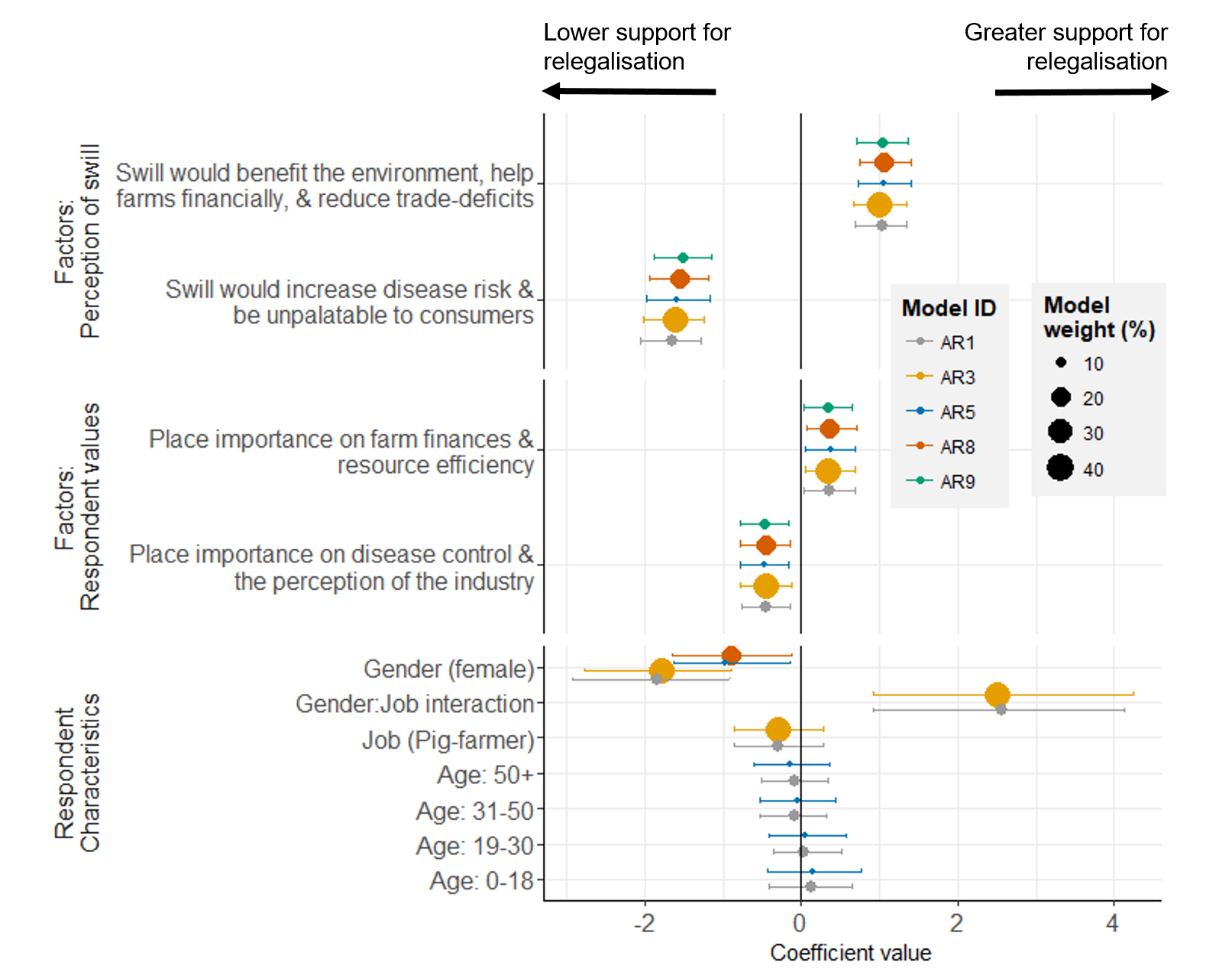


Fig J. Predictors of the support for the relegalisation of swill, among all respondents (n=163). The estimates plotted are from the five models with the greatest weighting (85% of model weight), where different colours are used for each model and model weights are proportional to the size of the points. Error bars are 89% credible intervals.

# Models of farmer support for the relegalisation of swill

The structure and priors used for the maximal model (model FS1) are described below. The predictors included in subsequent models are described in S2 Appendix Table 1; these models were fit using the same priors.

**Model FS1:**

**Model structure**

$LikertScore \sim Ordered\left( \varphi\right)$ [likelihood]

$logit\left( \varphi_{k} \right)=\alpha_{k}+ \alpha_{AGE_{GROUP\left[ i \right]}}+\alpha_{FARM_{SIZE\left[ i \right]}}+\beta_{GENDER}+$ [cumulative link & linear model]

$\beta_{FMD_{EXP}}+\beta_{SWILL_{EXP}}+\beta_{FEED_{TECH}}+$ […continued]

$\beta_{VAL\_F1}+ \beta_{VAL\_F2}+ \beta_{IMP\_F1}+ \beta_{IMP\_F2}$ […continued]

**Priors**

$\alpha_{k}=Normal(0,10)$ [common prior for each intercept]

$\alpha_{AGE\_GROUP\left[ a \right]}=Normal(0,\sigma_{A})$ [prior for age group intercepts]

$\alpha_{FARM\_SIZE\left[ F \right]}=Normal(0,\sigma_{F})$ [prior for farm size intercepts]

$(\beta_{GENDER},\beta_{FMD_{EXP}},\beta_{SWILL_{EXP}},\beta_{FEED_{TECH}}, \beta_{VAL_{F1}}, \beta_{VAL_{F2}}, \beta_{IMP_{F1}}, \beta_{IMP\_F2})=Normal\left( 0,10 \right)$ [Priors for slopes]

$(\sigma_{A},\sigma_{F})=HalfCauchy(0,1)$ [priors for $\sigma_{A},\sigma_{F}$]

Where,

$LikertScore$ is the score for the support for relegalisation of swill, (1-5, Definitely not – Definitely yes), amongst farmers (n=82).

$\alpha_{AGE\_GROUP\left[ a \right]}$ is the intercept for different age groups (shown in S2 Appendix Fig 1).

$\alpha_{FARM\_SIZE\left[ i \right]}$ is the intercept for different farm sizes (shown in S2 Appendix Fig 2).

$\beta_{GENDER}$is the slope for the respondent’s gender (1=female, 0=male).

$\beta_{FMD_{EXP}}$is the slope for whether or not the farm was directly affected the 2001 foot-and-mouth outbreak (1=affected, 0=not affected).

$\beta_{SWILL\_EXP}$is the slope for whether or not the farm has previously used will (1=yes, 0=no).

$\beta_{FEED\_TECH}$is the slope for whether the farmer uses wet or dry feed (1=wet, 0=dry).

$\beta_{VAL_{F1}} \& \beta_{VAL_{F2}}$are the slope for the first and second factor loadings for respondent’s values.

$\beta_{IMP\_F1}\& \beta_{IMP\_F2}$ are the slope for the first and second factor loadings for respondent’s perception of the impacts of swill.

Table A - Models predicting support for the relegalisation of swill, amongst pig farmers (n=82).

|  | Predictors | | | | | | | |  |  |  | Model output | | |
| --- | --- | --- | --- | --- | --- | --- | --- | --- | --- | --- | --- | --- | --- | --- |
| Model | Age group intercepts | Farm size intercepts | Gender | FMD experience | Experience using swill | Feed technology | Values: 1st FL | Values: 2nd FL | Perception of swill: 1st FL | Perception of swill: 2nd FL |  | pWAIC | WAIC | Model weight |
| FS1 | Y | Y | Y | Y | Y | Y | Y | Y | Y | Y |  | 17.9 | 187.1 | 0.01 |
| FS2 | Y | - | Y | Y | Y | Y | Y | Y | Y | Y |  | 15.7 | 185.9 | 0.01 |
| FS3 | - | Y | Y | Y | Y | Y | Y | Y | Y | Y |  | 16.4 | 186.4 | 0.01 |
| FS4 | - | - | Y | Y | Y | Y | Y | Y | Y | Y |  | 13.6 | 183.9 | 0.03 |
| FS5 | - | - | - | Y | Y | Y | Y | Y | Y | Y |  | 12.5 | 182.1 | 0.08 |
| FS6 | - | - | Y | Y | - | Y | Y | Y | Y | Y |  | 12.3 | 182.2 | 0.08 |
| FS7 | - | - | Y | Y | Y | - | Y | Y | Y | Y |  | 12.3 | 184.8 | 0.02 |
| FS8 | - | - | Y | - | Y | Y | Y | Y | Y | Y |  | 12.1 | 182.1 | 0.08 |
| FS9 | - | - | Y | - | - | Y | Y | Y | Y | Y |  | 10.9 | 180.6 | 0.16 |
| FS10 | - | - | - | Y | - | Y | Y | Y | Y | Y |  | 11.1 | 180.1 | 0.21 |
| FS11 | - | - | - | - | Y | Y | Y | Y | Y | Y |  | 11.0 | 180.4 | 0.18 |
| FS12 | - | - | - | Y | Y | - | Y | Y | Y | Y |  | 11.1 | 183.0 | 0.05 |
| FS13 | - | - | Y | Y | - | - | Y | Y | Y | Y |  | 10.9 | 182.7 | 0.06 |
| FS14 | - | - | Y | - | Y | - | Y | Y | Y | Y |  | 10.9 | 184.8 | 0.02 |

“FL” is factor loading; WAIC is the widely applicable information criterion score; pWAIC is the number of effective parameters.


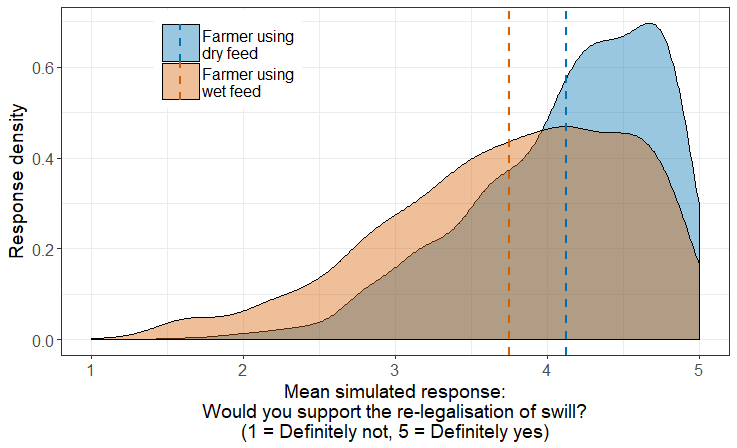


Fig K. Density plot of farmer support for the relegalisation of swill, comparing farmers who use wet or dry feed. The data are the mean simulated responses from 1000 respondents, based on model output from the three models with greatest weight (55% of model weight; S2 Appendix Table 1). The vertical lines designate the overall mean for each category of feeds.

# Models of farmer willingness to use swill, if it were relegalised

The structure and priors used for the maximal model (model WU1) are described below. The predictors included in subsequent models are described in S2 Appendix Table 2; these models were fit using the same priors.

**Model WU1:**

**Model structure**

$WillLikert \sim Ordered\left( \varphi\right)$ [likelihood]

$logit\left( \varphi_{k} \right)=\alpha_{k}+ \alpha_{AGE_{GROUP\left[ i \right]}}+\alpha_{FARM_{SIZE\left[ i \right]}}+\beta_{GENDER}+$ [cumulative link & linear model]

$\beta_{FMD_{EXP}}+\beta_{SWILL_{EXP}}+\beta_{FEED_{TECH}}+$ […continued]

$\beta_{VAL\_F1}+ \beta_{VAL\_F2}+ \beta_{IMP\_F1}+ \beta_{IMP\_F2}$ […continued]

**Priors**

$\alpha_{k}=Normal(0,10)$ [common prior for each intercept]

$\alpha_{AGE\_GROUP\left[ i \right]}=Normal(0,\sigma_{A})$ [prior for age group intercepts]

$\alpha_{FARM\_SIZE\left[ i \right]}=Normal(0,\sigma_{F})$ [prior for farm size intercepts]

$(\beta_{GENDER},\beta_{FMD_{EXP}},\beta_{SWILL_{EXP}},\beta_{FEED_{TECH}}, \beta_{VAL_{F1}}, \beta_{VAL_{F2}}, \beta_{IMP_{F1}}, \beta_{IMP\_F2})=Normal\left( 0,10 \right)$ [Priors for slopes]

$(\sigma_{A},\sigma_{F})=HalfCauchy(0,1)$ [priors for $\sigma_{A},\sigma_{F}$]

Where,

$WillLikert$is the score for the willingness to use swill, if it were relegalised, (1-5, Definitely not – Definitely yes), amongst farmers (n=82).

$\alpha_{AGE\_GROUP\left[ a \right]}$ is the intercept for different age groups (shown in S2 Appendix Fig 1).

$\alpha_{FARM\_SIZE\left[ i \right]}$ is the intercept for different farm sizes (shown in S2 Appendix Fig 2).

$\beta_{GENDER}$is the slope for the respondent’s gender (1=female, 0=male).

$\beta_{FMD_{EXP}}$is the slope for whether or not the farm was directly affected the 2001 foot-and-mouth outbreak (1=affected, 0=not affected).

$\beta_{SWILL\_EXP}$is the slope for whether or not the farm has previously used will (1=yes, 0=no).

$\beta_{FEED\_TECH}$is the slope for whether the farmer uses wet or dry feed (1=wet, 0=dry).

$\beta_{VAL_{F1}} \& \beta_{VAL_{F2}}$are the slope for the first and second factor loadings for respondent’s values.

$\beta_{IMP\_F1}\& \beta_{IMP\_F2}$ are the slope for the first and second factor loadings for respondent’s perception of the impacts of swill.

Table B - Models predicting farmer willingness to use swill, if it were relegalised (n=82).

|  | Predictors | | | | | | | |  |  |  | Model output | | |
| --- | --- | --- | --- | --- | --- | --- | --- | --- | --- | --- | --- | --- | --- | --- |
| Model  ID | Age group intercepts | Farm size intercepts | Gender | FMD experience | Experience using swill | Feed technology | Values: 1st FL | Values: 2nd FL | Perception of swill: 1st FL | Perception of swill: 2nd FL |  | pWAIC | WAIC | Model weight |
| WU1 | Y | Y | Y | Y | Y | Y | Y | Y | Y | Y |  | 17.1 | 248.5 | 0.00 |
| WU2 | Y | - | Y | Y | Y | Y | Y | Y | Y | Y |  | 14.9 | 245.4 | 0.00 |
| WU3 | - | Y | Y | Y | Y | Y | Y | Y | Y | Y |  | 15.0 | 246.2 | 0.00 |
| WU4 | - | - | Y | Y | Y | Y | Y | Y | Y | Y |  | 12.8 | 243.9 | 0.00 |
| WU5 | - | - | - | Y | Y | Y | Y | Y | Y | Y |  | 12.0 | 242.0 | 0.01 |
| WU6 | - | - | Y | Y | - | Y | Y | Y | Y | Y |  | 12.0 | 245.2 | 0.00 |
| WU7 | - | - | Y | Y | Y | - | Y | Y | Y | Y |  | 11.6 | 241.6 | 0.01 |
| WU8 | - | - | Y | - | Y | Y | Y | Y | Y | Y |  | 11.5 | 241.5 | 0.02 |
| WU9 | - | - | Y | - | - | Y | Y | Y | Y | Y |  | 10.7 | 243.5 | 0.01 |
| WU10 | - | - | - | Y | - | Y | Y | Y | Y | Y |  | 11.3 | 243.7 | 0.01 |
| WU11 | - | - | - | - | Y | Y | Y | Y | Y | Y |  | 10.5 | 239.2 | 0.05 |
| WU12 | - | - | - | Y | Y | - | Y | Y | Y | Y |  | 10.7 | 239.7 | 0.04 |
| WU13 | - | - | Y | Y | - | - | Y | Y | Y | Y |  | 10.7 | 243.1 | 0.01 |
| WU14 | - | - | Y | - | Y | - | Y | Y | Y | Y |  | 10.3 | 239.4 | 0.05 |
| WU15 | - | - | Y | - | - | - | - | - | Y | Y |  | 6.8 | 238.7 | 0.06 |
| WU16 | - | - | - | Y | - | - | - | - | Y | Y |  | 6.9 | 238.7 | 0.06 |
| WU17 | - | - | - | - | Y | - | - | - | Y | Y |  | 6.9 | 234.9 | 0.42 |
| WU18 | - | - | - | - | - | Y | - | - | Y | Y |  | 6.8 | 238.5 | 0.07 |
| WU19 | - | - | - | - | - | - | - | - | Y | Y |  | 5.7 | 236.6 | 0.18 |

“FL” is factor loading; WAIC is the widely applicable information criterion score; pWAIC is the number of effective parameters.


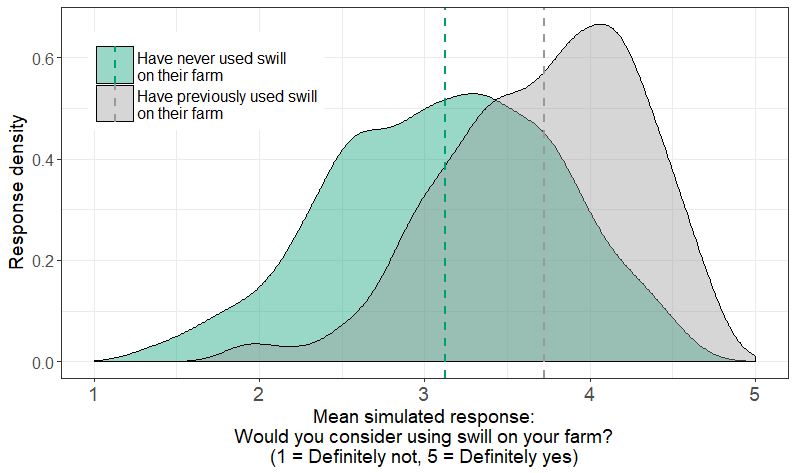


Fig L. Density plot comparing the effect of a respondent having previous experience of using swill on their willingness to use swill, if it were relegalised. The data are the mean simulated responses from 1000 respondents (controlling for other factors), based on model output from the model with highest weight (model WU17; S2 Appendix Table 2).

# References cited in S2 Appendix

1. Eurostat database. Agriculture statistics [Internet]. 2014 [cited 9 Jan 2014]. Available: http://ec.europa.eu/eurostat

2. Andreoletti O, Budka H, Buncic S, Colin P, Collins JD, De Koeijer A, et al. Opinion of the Scientific Panel on Biological Hazards on a request from the European Parliament on Certain Aspects related to the Feeding of Animal Proteins to Farm Animals. EFSA J. 2007; 1–41.
